# Recovering knot placements in Bayesian piecewise growth models with missing data

**DOI:** 10.3758/s13428-025-02716-0

**Published:** 2025-06-18

**Authors:** Ihnwhi Heo, Fan Jia, Sarah Depaoli

**Affiliations:** Department of Psychological Sciences, University of California, Merced, 5200 N. Lake Road, Merced, CA 95343 USA

**Keywords:** Bayesian estimation, Knot, Missing data, Piecewise growth, Prior distribution

## Abstract

Bayesian piecewise growth models (PGMs) are useful tools to capture nonlinear trends comprised of distinct developmental phases. An important parameter in Bayesian PGMs is the knot location – the time at which transitions arise between phases. While researchers can specify knot locations when they are known a priori, a more flexible approach is to estimate knot locations based on data. The Bayesian estimation of knot locations is largely affected by prior distributions and missing data; however, little is known about the impact of these two factors in recovering knot placements. In the current article, we conducted a Monte Carlo simulation study to examine the impact of different prior specifications and the presence of missing data on the recovery of knot placements in Bayesian PGMs. Simulation results indicated that in small sample sizes, knot location estimates were dictated by prior distributions. Even with larger sample sizes, the estimates remained sensitive to informative and inaccurate prior specifications. The presence of missing data complicated the recovery linked to certain priors. While negative consequences, such as bias in parameter estimates, were caused by a larger amount of missing data, this could be alleviated by informative and accurate priors. These findings emphasize the critical role and intertwined influence of prior distributions and missing data in reaching conclusions about changepoints. We present an illustrative example using real data with missing values to demonstrate the Bayesian estimation of knot locations under realistic scenarios. Recommendations for applied researchers are discussed.

Many social and behavioral scientists ask research questions surrounding how individuals or other research units change over time. Researchers are then to choose appropriate longitudinal statistical models to answer such questions. The latent growth model is a versatile statistical modeling framework that has its roots in structural equation modeling. Researchers use latent growth models to analyze intraindividual changes over time as well as interindividual differences in their patterns of change by postulating relationships between observed data and a latent continuum (Bollen & Curran, 2006; Grimm et al., 2016; Meredith & Tisak, 1990).

While many traditional latent growth models assume overall uninterrupted and smooth growth curves, it is not uncommon to expect growth trajectories that comprise separate developmental phases. The latent piecewise growth model (PGM) is a special type of latent growth model for analyzing nonlinear dynamic change processes consisting of distinct growth phases. For instance, Chung et al. (2017) fitted a PGM to examine a segmented growth trajectory of self-esteem from childhood to adulthood. In a similar vein, Wu et al. (2008) utilized a PGM to study how retention in the first grade influenced a growth shape in children’s mathematics and reading achievement. The utility of PGMs in incorporating segmented growth stages is appealing and thus has led to wide applications in substantive research (e.g., Hardy & Thiels, 2009; Jaggars & Xu, 2016; Lee & Rojewski, 2009; Patrick & Schulenberg, 2011).

However, the estimation of piecewise trends can be challenged by low convergence rates due to model complexity (Diallo et al., 2013; Kohli et al., 2015). The Bayesian estimation framework can be eminently suited to improve accuracy in estimating growth models by incorporating prior knowledge (Depaoli & Boyajian, 2014; Depaoli, 2013; Depaoli & Boyajian, 2014; Smid et al., 2020; Serang et al., 2015), even when the sample size is small (McNeish, 2016b). A perusal of the literature indicates that the Bayesian approach has been applied to estimate PGMs, particularly models with mixture components (Lock et al., 2018; Kohli et al., 2015). In addition, Peralta et al. (2022); Wang and McArdle (2008) implemented the Bayesian approach to piecewise mixed-effects modeling, which is based on a multilevel modeling framework. Simulation studies have been used to examine the performance of these Bayesian approaches. Kohli et al. (2015) demonstrated the advantages of Bayesian estimation in accurately recovering parameters of piecewise growth mixture models, while also highlighting its computational feasibility. Similarly, results from Wang and McArdle (2008) suggested that Bayesian methods produced reliable estimates and were successful in recovering parameter values for piecewise mixed-effects models. These studies underscore the potential benefits of the Bayesian approach to PGMs, paving the way for future methodological explorations.

A parameter that deserves attention in PGMs is the knot location – the time at which transitions arise between stages. Importantly, placing knots is a major issue in piecewise growth modeling. Researchers can specify single or multiple knots when their locations are known a priori based on theories or research designs to mark substantively important moments (Flora, 2008; Marvin et al., 2023; Sterba, 2014). More common scenarios are situations in which knot locations are unknown when researchers have limited knowledge about them. Under these scenarios, the misspecification of knot locations can backfire and lead to misleading research conclusions (Depaoli et al., 2023; Heo et al., 2024; Leite & Stapleton, 2011; Ning & Luo, 2017). An alternative approach is to freely estimate knot locations based on collected data (Harring et al., 2006; Kohli & Harring, 2013; Kwok et al., 2010; Ning & Luo, 2017). For example, Kohli and Sullivan (2019) fitted piecewise growth mixture models to estimate locations of knots in a trajectory of mathematics achievements from kindergarten to the eighth grade. While locating knots should be theoretically justified (Kwok et al., 2010; Marcoulides, 2018), it is a viable option to estimate the turning points and optimally represent a piecewise functional form of change (Kwok et al., 2010; Ning & Luo, 2017).

One important feature in Bayesian inference is a prior distribution: The information from prior distributions is combined with the likelihood of data to form posterior distributions. This characteristic implies that different prior specifications can influence study results, which have been extensively studied by previous literature in latent growth modeling (Depaoli, 2013, 2014; Depaoli & Boyajian, 2014; McNeish, 2016a; Winter & Depaoli, 2022). As members of the latent growth model family, PGMs are anticipated to produce varied outcomes, contingent on the selection of priors, especially the prior distributions for knot locations. Thus far, extant studies have focused on prior conditions within specific contexts under complete data scenarios, leaving the examination of a comprehensive range of prior specifications underexplored. Lock et al. (2018) considered specifying priors for the hyperparameters of truncated normal distributions in estimating multiple random knot locations for each individual. Specifically, they used noninformative uniform prior distributions for the mean hyperparameters of knot locations. For the variance hyperparameters, they considered uniform priors as well as scaled or unscaled half-Cauchy priors, as demonstrated in the supplement from Lock et al. (2018). Another study by Kohli et al. (2015) estimated a knot location based on a noninformative uniform prior distribution. Within the context of piecewise mixed-effects modeling, Wang and McArdle (2008) estimated knot locations using a noninformative multivariate normal prior. Peralta et al. (2022), on the other hand, used a truncated normal distribution as a prior to estimate knot locations. Additionally, they conducted a prior sensitivity analysis. However, a uniform prior for knot locations was utilized only in a specific simulation scenario.

Furthermore, missing data are a ubiquitous challenge in longitudinal research, and studies using PGMs suffer from missing data (e.g., Hu et al., 2020; Lee & Rojewski, 2009; Li et al., 2001). The presence of missing data can cause bias in parameter estimation and damage the generalizability of the sample (Graham, 2009; Shi et al., 2021). In particular, when missing data mechanisms are not ignorable or missing data handling methods do not meet the assumptions that corresponding methods necessitate in the model fitting step, bias can be introduced. Encountering missing data in longitudinal research can be attributable to multiple reasons, particularly attrition of participants at the midway point (Nicholson et al., 2017; Twisk & de Vente, 2002). The issue of participant attrition leading to a limited number of observations poses a critical challenge in PGMs, potentially causing biased estimates of knot locations. Bayesian estimation addresses missing data via data augmentation, which iteratively samples missing values from conditional distributions (Lee, 2007; Tanner & Wong, 1987). The process of Bayesian estimation via data augmentation has been demonstrated in estimating models with missing data under complex data structures or different missing data mechanisms (e.g., Lee & Song, 2004; Lee & Tang, 2006; Song & Lee, 2002). However, a paucity of research has examined the degree to which Bayesian estimation, in the presence of missing data, can successfully recover knot placements. To accurately detect changepoints and draw meaningful substantive conclusions, it is critical to ascertain the effectiveness of Bayesian PGMs in successfully retrieving the true knot locations, particularly in situations involving missing data.

## Novel contributions

This study extends findings and incorporates insights from previous research, introducing novel contributions in several key areas. First, we build upon Wang and McArdle ’s (2008) suggestion of exploring the impact of informative prior distributions in estimating knot locations. As such, we consider the specification of prior distributions using not only uniform priors (e.g., Kohli et al., 2015) but also truncated normal priors (e.g., Peralta et al., Peralta et al. ) to vary levels of informativeness and accuracy under extensive simulation conditions. Importantly, we acknowledge the previous contributions of Lock et al. (2018), who specified hyperpriors for both the mean and variance hyperparameters in estimating multiple random knot locations for each individual. Their use of scaled or unscaled half-Cauchy priors for the variance hyperparameter, instead of uniform priors, highlighted the benefits of informative priors for model convergence and reducing bias. However, our approach differs by evaluating the sensitivity of estimates under distinct and separate prior settings, assigning values to the hyperparameters that more closely mirror the scenarios encountered in applied piecewise growth modeling research. By doing so, our study evaluates distinct prior settings in various forms across simulation and illustrative studies.

Second, we emphasize, in line with Depaoli et al. (2023), the importance of investigating missing data in Bayesian piecewise growth modeling. Despite its prevalence, the impact of missing data on the estimation of knot locations has been largely overlooked in previous studies (e.g., Kohli et al., 2015; Lock et al., 2018; Peralta et al., 2022; Wang & McArdle, 2008), representing a critical gap in the literature. Our study addresses this gap and provides a significant contribution to understanding how missing data can affect the robustness and accuracy of knot location estimates in Bayesian PGMs. We consider different amounts and patterns of missing data in our simulation design (e.g., Heo et al., 2024), which reflects a realistic amount and pattern of missing data as will be delineated in our illustrative example. We particularly consider a missing at random (MAR) mechanism since modern missing data handling techniques assume MAR (van Buuren, 2018), and MAR has been a common assumption in both methodological and applied research with latent growth modeling (e.g., Depaoli et al., 2023; Heo et al., 2024; Shi et al., 2021; Winter & Depaoli, 2022).

Third, our work is grounded within a latent growth ‘curve’ modeling context that is non-mixture and non-mixed-effects^1^. We have chosen this simple yet prevalent modeling framework to focus on an understudied aspect: how missing data influence the estimation of knot locations. Although it might be more realistic to extend the framework into more complex scenarios, such as mixtures, isolating the effects of missing data on knot locations before introducing additional complexities sets a baseline for research with more advanced models. As a result, our study serves as a foundational step in addressing missing data issues in a simpler PGM. This approach complements existing studies (Kohli et al., 2015; Lock et al., 2018; Peralta et al., 2022; Wang & McArdle, 2008), which primarily focused on model parameters, including knot locations, within mixture or mixed-effects frameworks but did not systematically examine how missing data might affect parameter recovery. Hence, by systematically investigating knot placements within the piecewise growth modeling framework that is widely applied in both methodological and applied works (see, e.g., Marvin et al., 2023), our study advances the understanding of how Bayesian PGMs perform when missing data exist. We believe the current investigation contributes to a comprehensive overview of knot location recovery, complements existing works, and informs subsequent extensions to more complex settings.

### Goals and organization of the current study

While the placement of knots in Bayesian PGMs holds substantive importance, current methodological research has rarely focused on two influential factors in the estimation of knot locations – prior distributions and missing data. The current study aims to fill this gap by estimating knot locations under various conditions. The objective of this paper is to examine the impact of different prior specifications and the presence of missing data on the recovery of knot placements in Bayesian PGMs via simulation, and to demonstrate practical applications through illustrative examples using real data.

We begin by explaining the Bayesian estimation framework in the context of PGMs. In the subsequent sections, we present the key literature on specifying prior distributions for knot locations and estimating latent growth models in the presence of missing data. This information provides the background and context for the current investigation. Next, we detail the design of the simulation study, which evaluates the impact of different prior distributions and the presence of missing data on the recovery of knot placements. We provide details on the conditions and other settings for conducting the simulation study, and then we present the results. We further present an illustrative example by estimating knot locations in real data containing missing values. We conclude with a discussion of the key findings, provide advice for researchers, and suggest directions for future research.

## Bayesian piecewise growth modeling

The piecewise growth modeling framework introduces knot placements as additional parameters for estimating stage-specific growth rates. With a single knot, longitudinal changes are segmented into two distinct pieces. Adding multiple knots extends the division in a way that more than two developmental stages are described. As a consequence, knots increase the model complexity tied to the identification of PGMs (Bollen & Curran, 2006; Flora, 2008). In addition, the estimation of nonlinear growth trends, such as piecewise growth curves, may face issues such as nonconvergence or convergence on inadmissible parameter solutions, particularly with small sample sizes (Diallo et al., 2013). To address these methodological challenges, the Bayesian approach to piecewise growth modeling can be an alternative. By incorporating prior knowledge into statistical inference, the Bayesian estimation framework mitigates problems related to non-converged or inadmissible parameter estimates (Can et al., 2015; Kohli et al., 2015). The Bayesian scheme can aid in accurately estimating parameters of growth models with nonlinear trends, including piecewise growth trajectories (Kohli et al., 2015; Wang & McArdle, 2008) with mixture components or multiple knots (Lock et al., 2018). Additional benefits of the Bayesian methods include efficiently managing computational challenges associated with large parameter spaces and the inherent nonlinearity of the models (Kohli et al., 2015).

The Bayesian approach is applicable to PGMs with varying degrees of complexity. The simplest form of a PGM is the linear-linear model, which comprises two separate linear segments, connected by a single knot. This basic form of PGM has been widely studied in the methodological literature (Depaoli et al., 2023; Heo et al., 2024; Kohli & Harring, 2013; Kohli et al., 2015; Kwok et al., 2010; Leite & Stapleton, 2011) with many applications in applied research (Hu et al., 2020; Jaggars & Xu, 2016; Kohli & Sullivan, 2019). Due to the broad relevance and applicability of the linear-linear PGM with a single knot, we have chosen this PGM as the focal model in our current study. However, it is important to note that this simple PGM can be extended into more complex formats, including models with multiple knot locations, disjointed knots, individually varying knots, and others (e.g., Chung et al., 2017; Cudeck & Codd, 2012; Flora, 2008; Harring et al., 2021; Heo et al., 2024; Kroese et al., 2014; Lock et al., 2018). Next, we provide a brief overview of the Bayesian estimation framework for linear-linear PGMs with one single knot.

### Likelihood model

The likelihood model for a PGM can be defined within a structural equation modeling framework (Grimm et al., 2016). The common factor model and the latent factor scores for the PGM can be expressed as follows:

1 $$\begin{aligned} \begin{aligned} \varvec{y}_{i}&= \varvec{\Lambda }\varvec{\eta }_{i} + \varvec{\epsilon }_{i} \; \text {with} \; \varvec{\epsilon }_{i} \sim \mathcal {N}(\varvec{0}, \varvec{\Omega }_{\varvec{y}}),\\ \varvec{\eta }_{i}&= \varvec{\alpha } + \varvec{\zeta }_{i} \;\,\,\,\, \text {with} \; \varvec{\zeta }_{i} \sim \mathcal {N}(\varvec{0}, \varvec{\Omega }_{\varvec{\eta }}). \end{aligned} \end{aligned}$$

In Eq. (1), $\varvec{y}_{i}$ is a $J \times 1$ vector that contains repeated outcome measures for individual *i* where *J* equals the number of repeated measurements, $\varvec{\Lambda }$ refers to a $J \times M$ matrix of factor loadings, $\varvec{\eta }_{i}$ is an $M \times 1$ vector of growth factors for individual *i*, $\varvec{\epsilon }_{i}$ refers to a $J \times 1$ vector of error terms that cannot be accounted for by the trajectory for individual *i*, $\varvec{\Omega }_{\varvec{y}}$ refers to a *J*-dimensional covariance matrix, $\varvec{\alpha }$ is an $M \times 1$ vector that stores growth factor means, $\varvec{\zeta }_{i}$ is an $M \times 1$ vector of deviations from the mean for individual *i*, and $\varvec{\Omega }_{\varvec{\eta }}$ is an *M*-dimensional covariance matrix.

When formulating PGMs, the dimensions and elements of the $\varvec{\Lambda }$ matrix are adjusted to specify piecewise growth trajectories and knot locations. Specifically, the number of rows in $\varvec{\Lambda }$ corresponds to the number of repeated measurements *J*, while the number of columns corresponds to the growth factors *M*, and elements of the matrix are adjusted to locate knots. For a linear-linear PGM, at least five time points of repeated measurements are required to identify the model (Bollen & Curran, 2006; Flora, 2008).

Suppose we have data from seven equidistant time points, and we place a knot at the fourth time point. In this scenario, the $\varvec{\Lambda }$ matrix is a $7 \times 3$ matrix because we have seven repeated measurements and three growth factors (i.e., latent intercept, first latent linear slope, and second latent linear slope). The first column consists of “1”s because the intercept remains constant over time. The second and third columns represent the hypothesized patterns of the piecewise growth curve. Typically, for slope factors, the value at the first time point is set to 0. As a result, the knot location at the fourth time point is coded as 3. Thus, the $\varvec{\Lambda }$ matrix is specified as follows:

2 $$\begin{aligned} \varvec{\Lambda } = \begin{bmatrix} 1 & 0 & 0 \\ 1 & 1 & 0 \\ 1 & 2 & 0 \\ 1 & 3 & 0 \\ 1 & 3 & 1 \\ 1 & 3 & 2 \\ 1 & 3 & 3 \\ \end{bmatrix}. \end{aligned}$$

The second column holds the loadings of the first linear slope factor and captures the rate of change in measurements leading up to the knot location at the fourth time point. The third column defines the loadings of the second linear slope factor and captures the rate of change in measurements following the knot location. Note that the pre-knot slope does not affect measurements after the knot locations, and similarly, the post-knot slope does not influence measurements before the knot location. A more general form of the $j^{\text {th}}$ row in the $\varvec{\Lambda }$ matrix can be expressed in the following manner (Grimm et al., 2016):

3 $$\begin{aligned} \varvec{\Lambda } = [1 \,\,\, \text {min}(t_j, \gamma ) \,\,\, \text {max}(t_j - \gamma , 0)], \end{aligned}$$

where $t_j$ represents the time metric associated with occasion *j* with $1 \le j \le J$ (e.g., $t_1$ equals 0 because the first time point is coded as 0), $\gamma$ is the knot location (e.g., $\gamma$ equals 3 when the fourth time point is the true knot location), and the dimension of the $\varvec{\Lambda }$ matrix is $J \times 3$. Equation (3) is a generalized expression of a linear-linear PGM with a single knot. To formulate PGMs with nonlinear slopes or more than one knot location, readers are referred to Grimm et al. (2016); Harring et al. (2021).

### Prior distributions

Specifying prior distributions is essential for implementing Bayesian inference in PGMs. We consider four sets of parameters with their respective prior specifications. The first set of parameters to consider is the latent factor means. We typically assume that the means of growth factors follow normal distributions:

$$\begin{aligned} {\begin{matrix} \alpha & \sim \mathcal {N}(\mu _{\alpha }, \sigma _{\alpha }^{2}), \\ \end{matrix}} \end{aligned}$$

where $\alpha$ is the mean of growth factors (i.e., latent means and slope factors), $\mu _{\alpha }$ is the mean hyperparameter that indicates the center of this prior distribution, and $\sigma _{\alpha }^2$ is the variance hyperparameter that determines the informativeness of this prior distribution.

The second parameter is the factor covariance matrix $\varvec{\Omega }_{\varvec{\eta }}$. It is common to use the inverse Wishart distribution as the prior for this parameter:

$$\begin{aligned} \varvec{\Omega }_{\varvec{\eta }} \sim \mathcal{I}\mathcal{W}(\varvec{\Psi }, \nu ), \end{aligned}$$

where $\varvec{\Psi }$ is a positive definite matrix, and $\nu$ represents the degrees of freedom that determine the informativeness of this prior.

The third set of parameters is the variance of error terms in $\varvec{\Omega _{\varvec{y}}}$. Typically, we map an inverse gamma distribution to each element of the $\varvec{\Omega _{\varvec{y}}}$ matrix:

$$\begin{aligned} \sigma _{\epsilon _{ij}}^{2} \sim \mathcal{I}\mathcal{G}(a, b), \end{aligned}$$

where $\epsilon _{ij}$ is the error term of individual *i* at time point *j*, and *a* and *b* are the shape and scale hyperparameters, respectively.

The fourth and last parameter is the knot location $\gamma$. We introduce two options for prior distributions on $\gamma$. One option is to use the uniform distribution to indicate a diffuse prior setting over the knot location:

$$\begin{aligned} \gamma \sim \mathcal {U}(\text {min}(t_j), \text {max}(t_j)), \end{aligned}$$

where $\text {min}(t_j)$ and $\text {max}(t_j)$ are the initial and last time points, respectively. Specifying uniform prior distribution indicates that all values for $\gamma$ are equally likely, making this prior specification noninformative. The other option is to implement a truncated normal distribution to leverage different degrees of informativeness and accuracy with respect to knot location:

$$\begin{aligned} \gamma \sim \mathcal {N}(\mu _\gamma , \sigma _{\gamma }^{2}) \, \mathcal {T}(\text {min}(t_j), \text {max}(t_j)), \end{aligned}$$

where $\mu _\gamma$ defines the center of this prior distribution, $\sigma _{\gamma }^{2}$ defines the informativeness of the prior distribution via the distribution’s variance, and $\mathcal {T}(\text {min}(t_j), \text {max}(t_j))$ indicates that this normal prior distribution is truncated, ranging from the initial to the last time points.

### Posterior inference

Bayesian posterior inference combines the likelihood and the prior distribution to update prior knowledge and obtain the posterior distribution. This updating process is based on the tenet called Bayes theorem:

4 $$\begin{aligned} p(\varvec{\theta }|\varvec{y}_i) = \frac{ p(\varvec{y}_i|\varvec{\theta }) p(\varvec{\theta })}{p(\varvec{y}_i)}, \end{aligned}$$

where $\varvec{\theta }$ is a vector of parameters. In Eq. (4), the prior distribution, $p(\varvec{\theta })$, that reflects our prior knowledge about parameters is updated to the posterior distribution, $p(\varvec{\theta }|\varvec{y}_i)$, that reflects our posterior knowledge about parameters, by incorporating the likelihood, $p(\varvec{y}_i|\varvec{\theta })$, that represents the information from the sample data. The denominator on the right side of the equation, $p(\varvec{y}_i)$, is the marginal likelihood that serves as a normalizing constant in the Bayesian estimation framework.

The analytic derivation of the posterior distribution can be difficult. An alternative approach is to simulate draws from the posterior distribution to approximate it by employing an iterative sampling scheme called a Markov chain Monte Carlo (MCMC) algorithm (Gamerman & Lopes, 2006; Robert & Casella, 2004). This study uses the R rjags package that integrates JAGS into the R environment, and JAGS uses the Gibbs sampler as an MCMC algorithm (Plummer et al., 2003; Plummer, 2016). The Gibbs sampler draws posterior samples from conditional distributions in an iterative fashion (Geman & Geman, 1984). Suppose that the parameter vector, $\varvec{\theta }$, is comprised of *p* parameters: $\varvec{\theta }^{(s)}=(\theta _{1}^{(s)}, \theta _{2}^{(s)}, \ldots , \theta _{p}^{(s)})$, where the superscript within the parenthesis refers to the $s^{\text {th}} = 1, 2, \ldots , S$ iteration. To implement the Gibbs sampler, a set of initial values of each parameter, $\varvec{\theta }^{(0)}$, is required, which can be defined by researchers or randomly generated by the package. A general procedure of the Gibbs sampling is described as follows:

5 $$\begin{aligned} {\begin{matrix} \theta _{1}^{(s+1)} & \sim p(\theta _{1}|\theta _{2}^{(s)}, \theta _{3}^{(s)}, \ldots , \theta _{p}^{(s)}, \varvec{y}_i), \\ \theta _{2}^{(s+1)} & \sim p(\theta _{2}|\theta _{1}^{(s+1)}, \theta _{3}^{(s)}, \ldots , \theta _{p}^{(s)}, \varvec{y}_i), \\ \theta _{3}^{(s+1)} & \sim p(\theta _{3}|\theta _{1}^{(s+1)}, \theta _{2}^{(s+1)}, \ldots , \theta _{p}^{(s)}, \varvec{y}_i), \\ & \vdots \\ \theta _{p}^{(s+1)} & \sim p(\theta _{p}|\theta _{1}^{(s+1)}, \theta _{2}^{(s+1)}, \ldots , \theta _{p-1}^{(s+1)}, \varvec{y}_i), \end{matrix}} \end{aligned}$$

where at each $(s+1)^{\text {th}}$ iteration, posterior samples are alternately drawn from the conditional distribution using parameter values at the $s^{\text {th}}$ iteration. A collection of posterior samples is referred to as a Markov chain, which can be denoted as $\{\varvec{\theta }^{(1)}, \varvec{\theta }^{(2)}, \ldots , \varvec{\theta }^{(s)}, \ldots , \varvec{\theta }^{(S-1)}, \varvec{\theta }^{(S)}\}$. However, samples drawn at the initial iteration such as $\{\varvec{\theta }^{(1)}, \varvec{\theta }^{(2)}, \varvec{\theta }^{(3)}, \ldots \}$ are not stable to represent the target posterior distribution; hence, an initial set of posterior samples is discarded from a chain. These discarded samples are referred to as burn-in or warm-up samples. The remaining posterior samples are used for subsequent analysis.

In practice, researchers may run multiple Markov chains in a parallel manner, using different sets of initial values. After the burn-in samples are discarded, the posterior samples from these chains should, in principle, converge and fully approximate the target posterior distribution. It is thus important to evaluate whether chains have converged using convergence diagnostics. A common convergence diagnostic in previous literature in Bayesian piecewise growth modeling (e.g., Depaoli et al., 2023; Heo et al., 2024; Lock et al., 2018) is the $\widehat{R}$ statistic. It measures the potential scale reduction of parameter estimates when multiple chains are used in the MCMC process and compares the within-chain variability to between-chain variability. When multiple chains are converged, the $\widehat{R}$ statistics are close to 1. In addition, visual tools such as trace plots are also used to evaluate convergence. In trace plots, convergence is evidenced by multiple chains that are mixed and thus indistinguishable from each other. After determining chain convergence, the posterior distributions are summarized by calculating summary statistics such as the posterior mean, standard deviation, and 95% credible intervals.

#### Bayesian inference with missing data

In Bayesian inference with missing data, the observed data are augmented with missing data, which are treated as unknown parameters – a concept referred to as data augmentation (Tanner & Wong, 1987). In this case, the missing data are naturally filled in. To illustrate the data augmentation method, a brief description of the three missing data mechanisms is necessary (Little & Rubin, 2002; Schafer & Graham, 2002; van Buuren, 2018). First, data are missing completely at random (MCAR) when the probability of being missing is independent of both observed and missing values of variables in the dataset. Second, data are missing at random (MAR) when the probability of being missing depends on observed values but not on missing values. Lastly, data are said to be missing not at random (MNAR) when the probability of being missing is dependent on missing values, even after controlling for observed values. MNAR data are referred to as nonignorable missingness and can cause bias in parameter estimates (Graham, 2009; Kristman et al., 2004).

In Bayesian inference via data augmentation, the Gibbs sampling in Eq. (5) is expanded to sample not only the unknown parameter values but missing values from the conditional distribution. Therefore, the missing data are iteratively sampled at each iteration *s* from their conditional distributions. The complete likelihood is subsequently combined with the prior distribution to derive the posterior distribution in Eq. (4). Readers can consult Daniels and Hogan (2008); Gelman et al. (2013); Lee (2007) for an extensive exposition of data augmentation in Bayesian posterior inference under three different missing data mechanisms (Little & Rubin, 2002; Rubin, 1976; Schafer & Graham, 2002; van Buuren, 2018).

## Specifying prior distributions for knot placement

Recovering knot locations is crucial for drawing conclusions about transitions in longitudinal trajectories. As prior distributions are a key factor that influences the Bayesian estimation of knot locations, specifying priors is an essential task. Previous approaches to specifying prior distributions for knot locations have focused on specific prior settings under complete data situations, primarily within the frameworks of piecewise growth mixture modeling and mixed-effects modeling. We briefly peruse these studies with an emphasis on prior specifications.

Lock et al. (2018) estimated multiple random knot locations for each individual in two-class piecewise growth mixture models with linear trajectories. To specify priors that are robust to the scales of variables and generally applicable, they implemented hyperpriors for the mean and variance hyperparameters of truncated normal distributions. For the mean hyperparameter, they used the uniform distribution. For the variance hyperparameter, they used the uniform distribution but also compared it with scaled or unscaled half-Cauchy priors in the supplement. Given the nature of their work, where multiple random knot locations were present for individuals, the hyperpriors were set not to cover the full range of time points to circumvent model identifiability issues at multiple boundaries. The results generally indicated successful recovery of true knot locations. However, in conditions with lower class proportions for one class compared with the other class, the estimates were slightly overestimated.

A simpler model with single knots was examined in Kohli et al. (2015). Their simulation study used a two-class linear-linear piecewise growth mixture model as the population model. They specified a uniform prior distribution for knot locations across each latent class where the prior covered all time points. The results overall indicated a negligible level of average bias in the knot location estimates. However, under specific simulation conditions where the knot locations for both classes were close to each other, convergence rates were found to be poor.

We now turn to a study by Peralta et al. (2022), who utilized a bivariate linear-linear piecewise mixed-effects model. For the priors on knot locations, they specified a truncated normal distribution for each outcome variable, with the mean hyperparameter set at the midpoint of the time scale and the variance hyperparameter fixed at a quarter of the time scale. Here, the normal distribution was truncated between the minimum and maximum time points to ensure that plausible knot location values fell within the time frame. The study reported successful parameter recovery, including knot locations across most of the conditions. However, under conditions of medium sample size and low association between the two outcome variables, it faced nonconvergence issues and observed relative bias exceeding 10% in the variance estimates of knot locations.

In a further analysis, Peralta et al. (2022) conducted a prior sensitivity analysis under one specific simulation condition where the sample size was small, the association of error terms was medium, and the association between the bivariate variables was high. An alternative prior setting for knot locations was a uniform distribution. They found that parameter estimates were comparable to those obtained with the truncated normal prior, albeit with longer computation times.

We could review that previous studies focused on single specific prior settings for estimating knot locations or considered an alternative set of priors only for a limited simulation scenario, and the models examined were piecewise growth mixture models or piecewise mixed-effects models. However, our examination explores various prior specifications across a wide range of simulation conditions within the context of non-mixture PGMs and is thus comprehensive. Yet, missing data is another consideration in estimating PGMs, and we peruse literature relevant to our investigation in the next section.

## Estimating growth models with missing data

Attrition can create problems in producing accurate parameter estimates and obtaining samples that fully represent the true population (Graham, 2009). As a result, it is important to understand the impact of attrition on estimating PGMs. There exists a notable gap in the current literature regarding the exploration of Bayesian parameter estimation with missing data in PGMs. While one study has focused on model evaluation of Bayesian PGMs under attrition scenarios (Heo et al., 2024), this specific aspect remains unexplored. Nonetheless, alternative approaches for handling missing data have been investigated within the context of maximum likelihood estimation in linear latent growth models. We outline and discuss their respective findings as follows.

Shi et al. (2021) examined the impact of attrition in the context of small sample sizes with non-normally distributed data. They generated datasets with both MCAR and MAR mechanisms and imposed 10%, 20%, or 30% of missingness levels. When the data were MCAR, parameter estimates closely approximated the true values, regardless of the other simulation conditions. When the data were MAR, however, biased parameter estimates were obtained when the proportion of missing data was 30%. The degree of bias was larger for the variances and covariances of the growth factors than for the means of the growth factors.

**Fig. 1:**
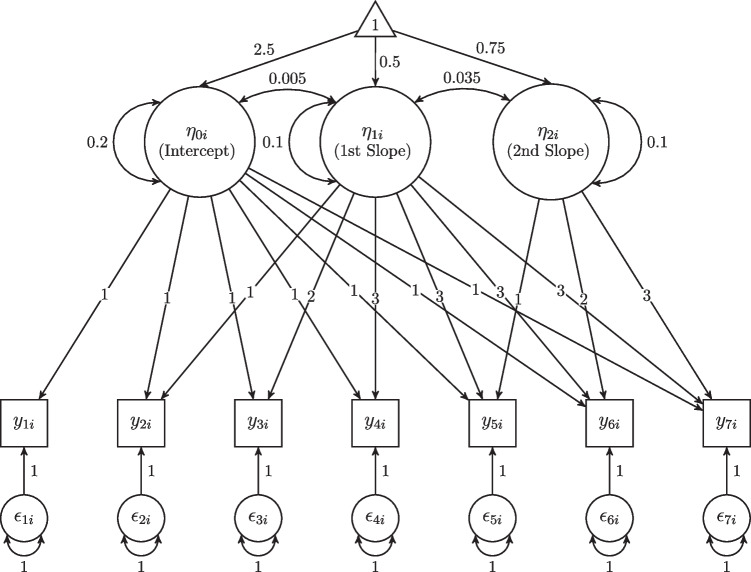
A path diagram for the population model

Bias resulting from attrition was also demonstrated in Nicholson et al. (2017). They considered MCAR, MAR, MNAR, and a combination of the three mechanisms, with 25% as the proportion of missing data. In addition, parameter recovery was evaluated under different missing data handling techniques: listwise deletion, full information maximum likelihood (FIML) with no auxiliary variable, FIML with an unrelated auxiliary variable, and FIML with a related auxiliary variable. Their findings revealed that, except for the MCAR condition and when a related auxiliary variable was included in the analysis model, bias was observed in the latent mean estimates of the slope factor. Including a related auxiliary variable mitigated the bias even for the MNAR and combination mechanisms conditions. The inclusion of the unrelated auxiliary variable did not worsen the estimation accuracy compared with conditions where no auxiliary variable was used. Among the four missing data mechanisms, the largest bias was observed under the MNAR data.

Although the two previous studies were not based on Bayesian estimation, and their models were not PGMs, they highlighted the adverse impact of attrition on parameter estimation under various missing data mechanisms. Our investigation focused on the Bayesian estimation of knot locations in the presence of MAR data to better understand the recovery of knot locations. Details of the simulation setup are provided next.

## Simulation design

We carried out a Monte Carlo simulation study to evaluate how different prior specifications and the presence of missing data impact the recovery of knot locations in Bayesian PGMs. We designed the simulation study, where all the levels of the three following factors were fully crossed: sample sizes (three levels), missing data (seven levels), and prior specifications (seven levels). This design resulted in a total of 147 cells. Next, we provide a detailed explanation of each design factor considered in the simulation setup. We also describe the population model, settings for Bayesian estimation, and outcome measures. Reproducible materials, including simulated data and annotated R code and JAGS syntax, are available as online supplementary materials at https://osf.io/j46bf/.

### Population model

The population model for the true data-generating mechanism was a linear-linear PGM with seven equidistant repeated outcome measures. The true knot location was set at the fourth time point (see Eq. (2)). Figure 1 presents a path diagram for the population model. The population parameter values were determined based on Depaoli et al. (2023); Heo et al. (2024), and this choice allowed us to examine the model’s sensitivity to detecting knot locations under missing data conditions while maintaining its generalizability (Kwok et al., 2010; Ning & Luo, 2017). The mean of the second linear slope after the first linear slope reflects a large change in terms of standardized effect size computation (Cohen, 2002; Kwok et al., 2010; Raudenbush & Liu, 2001).

### Sample size

Sample size plays an important role in Bayesian inference. For instance, observed data from small sample sizes convey relatively less information, and in turn, this leads to a stronger influence of prior distributions on posterior inference. Hence, we considered levels of the sample size condition to assess the relative strength of the priors in relation to sample sizes that are routinely encountered in applied research, as well as those examined in previous methodological studies (Depaoli et al., 2023; Heo et al., 2024; Kohli et al., 2015; Wang & McArdle, 2008; Winter & Depaoli, 2022). To this end, we considered three levels of the sample size condition: $n =$ 50 (representing a small sample size), 150 (medium sample size), and 500 (large sample size).

### Missing data

For the missing data condition, we referred to Heo et al. (2024) to manipulate the pattern and amount of missing data. For the missing data pattern, we considered attrition patterns in which the presence of missing values at a specific occasion ensured missing values in subsequent occasions (Nicholson et al., 2017; Twisk & de Vente, 2002; Winter & Depaoli, 2022). To cover different attrition patterns, we used linear and quadratic functions to model the probability of attrition across seven repeated measurements (Ortega-Azurduy et al., 2008). A linear function was used to compute a constant increase in missing data proportion across repeated measurements. On the other hand, a quadratic function was used to provide either an accelerated increase (i.e., missing values concentrated at the end of the study) or a decelerated increase (i.e., missing values concentrated at the beginning of the study) in missing data proportion across repeated measurements. For the amount of missing data, we manipulated it based on the proportion of missing values at the last (i.e., seventh) time point. The specific proportions of missing values at the last time point were set at 0%, 30%, or 70% to align with typical levels of missing data observed in longitudinal research (Gustavson et al., 2012; Wu et al., 2016). These considerations resulted in seven levels of the missing data condition:

- No missing data (Complete).
- Decelerated increase of missing values with 30% in the end (Concen-S-30%).
- Constant increase of missing values with 30% in the end (Constant-30%).
- Accelerated increase of missing values with 30% in the end (Concen-E-30%).
- Decelerated increase of missing values with 70% in the end (Concen-S-70%).
- Constant increase of missing values with 70% in the end (Constant-70%).
- Accelerated increase of missing values with 70% in the end (Concen-E-70%).

#### Missing data generation

We generated MAR data by predicting the missingness on indicators from the second to the seventh time points through logistic regression (Agresti, 2012):

6 $$\begin{aligned} \text {log}\frac{p(y_{ij}\,\text {is missing})}{1-p(y_{ij}\,\text {is missing})}=b_{0,j} + b_1 z_{i1}. \end{aligned}$$

In Eq. (6), $z_{i1}$ is a standardized $y_{i1}$, $b_{0,j}$ is the intercept at occasion *j*, and $b_1$ is the regression weight. The probability of missingness from $y_{i2}$ to $y_{i7}$ was determined by the standardized score of the first indicator, thus satisfying the MAR assumption. We fixed the regression weight $b_1$ at 1.48 to establish a strong association between the cause of missingness and the probability of data being missed (e.g., Enders & Mansolf, 2018). To manipulate the proportions of missing data, we varied the intercept $b_{0,j}$. Specifically, the proportion of missing data at each time point ($t_j=0, 1, \ldots , 6$) was calculated using the following equations (see Fig. 3 for missing data patterns under these equations):

- For Concen-S-30%, $$\displaystyle -\frac{5}{6}(t_j-6)^2+30$$.
- For Constant-30%, $$5t_j$$.
- For Concen-E-30%, $$\displaystyle \frac{5}{6}t_j^2$$.
- For Concen-S-70%, $$\displaystyle -\frac{35}{18}(t_j-6)^2+70$$.
- For Constant-70%, $$\displaystyle \frac{35}{3}t_j$$.
- For Concen-E-70%, $$\displaystyle \frac{35}{18}t_j^2$$.

Using the $b_1$ value of 1.48 and the $b_{0,j}$ values at each occasion, we inserted $z_{i1}$ into the logistic regression model to obtain a vector of probabilities. These probabilities were subsequently used as success probabilities of a binomial distribution for generating missing data indicators. Next, we removed the existing values when the missing data indicators were 1, and otherwise, the existing values were retained. We repeated this process for each level of the missing data condition.

**Fig. 2:**
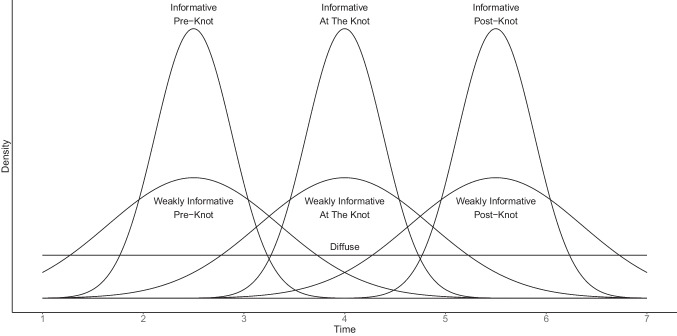
Prior specifications

**Fig. 3:**
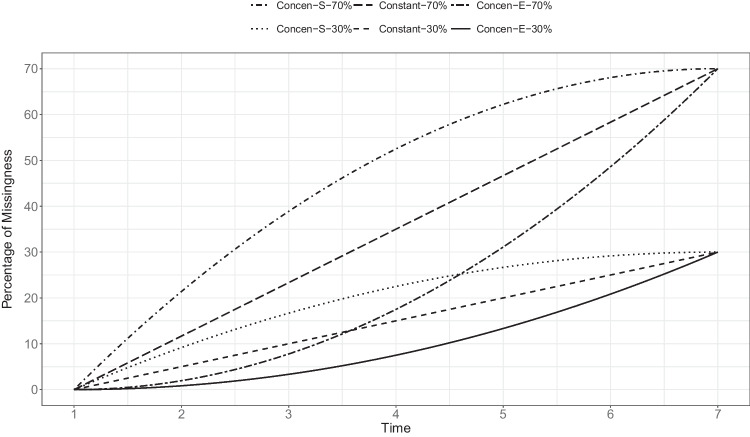
Missing data patterns

### Prior specification

We systematically manipulated the prior specification condition for knot locations using both a uniform distribution and a truncated normal distribution, which resulted in seven levels. All these levels are described in Fig. 2. The justifications for these specifications are provided below:

- $\gamma \sim \mathcal {U}(0, 6)$. This uniform distribution is a diffuse prior distribution (DIF), in which any values for knot locations from the first time point (coded as 0) to the seventh time point (coded as 6) are equally likely.
- $\gamma \sim \mathcal {N}(3, 0.146) \, \mathcal {T}(0, 6)$. This truncated normal distribution is an informative prior distribution located at the true knot location, which is at the fourth occasion (coded as 3). We call this prior specification the informative prior at the true knot location (I-ATK). The variance of 0.146 is determined in such a way that informative prior distributions overlap with each other by 10%.
- $\gamma \sim \mathcal {N}(3, 0.730) \, \mathcal {T}(0, 6)$. This truncated normal distribution is a weakly informative prior distribution located at the true knot location. We call this prior specification the weakly informative prior at the true knot location (WI-ATK). The variance of 0.730 is determined by multiplying 5 by the variance of the informative prior distribution.
- $\gamma \sim \mathcal {N}(1.5, 0.146) \, \mathcal {T}(0, 6)$. This truncated normal distribution is an informative prior distribution that is incorrectly located before the true knot location, which is at the time point 2.5 (coded as 1.5). We followed the Lock et al. ’s (2018) approach of using the midpoint to misplace the mean hyperparameter of knot locations. Hence, the incorrect location at the time point 2.5 is determined as a midpoint between the first time point and the true location. We call this prior specification the informative prior at the pre-knot location (I-PRK).
- $\gamma \sim \mathcal {N}(1.5, 0.730) \, \mathcal {T}(0, 6)$. This truncated normal distribution is a weakly informative prior distribution that is incorrectly located before the true knot location, which is at the time point 2.5. We call this prior specification the weakly informative prior at the pre-knot location (WI-PRK).
- $\gamma \sim \mathcal {N}(4.5, 0.146) \, \mathcal {T}(0, 6)$. This truncated normal distribution is an informative prior distribution that is incorrectly located after the true knot location, which is at the time point 5.5 (coded as 4.5). This incorrect location is determined as a midpoint between the true location and the last time point. We call this prior specification the informative prior at the post-knot location (I-PSK).
- $\gamma \sim \mathcal {N}(4.5, 0.730) \, \mathcal {T}(0, 6)$. This truncated normal distribution is a weakly informative prior distribution that is incorrectly located after the true knot location, which is at the time point 5.5. We call this prior specification the weakly informative prior at the post-knot location (WI-PSK).

For other parameters than knot locations, we referred to Kohli et al. (2015) and specified diffuse prior distributions in the following way:

- For the mean of the growth factors, the following normal prior distribution was used: $\mathcal {N}(0, 10^6)$.
- For the covariance matrix of the growth factors, the following inverse Wishart prior distribution was used: $\mathcal{I}\mathcal{W}(\varvec{I}_{3 \times 3}, 3)$, where $\varvec{I}_{3 \times 3}$ is a 3-dimensional identity matrix.
- For the residual variances, the following inverse gamma prior distribution was used: $\mathcal{I}\mathcal{G}(0.001, 0.001)$.

### Bayesian estimation

We generated 500 replications per cell using the R lavaan package (Rosseel, 2012) and used the rjags package in R (Plummer, 2016) to estimate parameters via the Bayesian estimation framework. For an MCMC algorithm, the Gibbs sampler was implemented with four chains each consisting of 25,000 iterations after discarding the first 5,000 iterations as the burn-in period. We thinned the chain by only retaining every $10^{\text {th}}$ sample to reduce high autocorrelations. We assessed chain convergence using $\widehat{R}$ statistics (Vehtari et al., 2021), with a cutoff value lower than 1.1^2^.

### Outcome measures

We used three numerical measures and one visual method to evaluate the recovery of knot placements. The numerical measures included coverage rate, average bias, and root mean squared error (RMSE). For the visual method, we created ridgeline plots to display distributions of posterior means for knot locations. Details of the way each numerical measure was calculated are explained:

- Coverage rate: proportions of converged replications in which a 95% credible interval from each converged replication contains the true parameter value of knot location:
  $$\begin{aligned} \text {Coverage rate} = \frac{1}{R_c}\sum _{s=1}^{R_c} I(\theta _{\gamma } \in [L_s, U_s]), \end{aligned}$$
  where $R_c$ is the number of converged replications, $\theta _{\gamma }$ is the true parameter value of knot location (i.e., $\theta _{\gamma }=3$ as the fourth time point is the knot location in the population model), $I(\cdot )$ is a 95% credible interval, and $L_s$ and $U_s$ are respectively a lower and an upper limit of an interval at the $s^{\text {th}}$ replication.
- Average bias: the mean of difference between parameter estimates of the knot location and its true parameter value over the converged replications:
  $$\begin{aligned} \text {Average bias} = \frac{1}{R_c} \sum _{s=1}^{R_c} (\hat{\theta }_{\gamma _{s}}-\theta _{\gamma }), \end{aligned}$$
  where $\hat{\theta }_{\gamma _{s}}$ is a parameter estimate of the knot location at the $s^{\text {th}}$ replication.
- RMSE: the square root of the mean of the squared differences between parameter estimates of the knot location and its true value over the converged replications:
  $$\begin{aligned} \text {RMSE} = \sqrt{\frac{\sum _{s=1}^{R_c}(\hat{\theta }_{\gamma _{s}}-\theta _{\gamma })^{2}}{R_c}}. \end{aligned}$$

## Simulation results

On average, 70.27% of replications had $\widehat{R}$ values for all parameters lower than 1.1, with a median of 81.00%. Convergence rates varied drastically across simulation conditions; hence, we conducted a thorough examination of convergence rates and reported the findings. As for the other outcome measures, we presented results only for replications upon successful convergence (Fig. 3).

**Table 1:** Convergence rate

	*n* = 50	*n* = 150	*n* = 500	*n* = 50	*n* = 150	*n* = 500	*n* = 50	*n* = 150	*n* = 500
	Complete			Concen-S-30%			Consen-S-70%		
DIF	0.150	0.414	**0.950**	0.088	0.350	**0.860**	0.050	0.168	0.536
I-ATK	**1.000**	**0.994**	**1.000**	**1.000**	**0.998**	**0.998**	**1.000**	**0.998**	**0.996**
WI-ATK	0.628	0.758	**0.984**	0.626	0.762	**0.972**	0.614	0.708	**0.854**
I-PRK	**0.870**	0.796	**0.978**	**0.854**	0.742	**0.930**	**0.852**	0.670	0.742
WI-PRK	0.270	0.484	**0.950**	0.256	0.406	**0.896**	0.196	0.256	0.626
I-PSK	**0.926**	**0.916**	**0.914**	**0.908**	**0.900**	**0.810**	**0.920**	**0.926**	**0.890**
WI-PSK	0.348	0.582	**0.938**	0.312	0.498	**0.864**	0.310	0.350	0.616
				Constant-30%			Constant-70%		
DIF				0.096	0.308	**0.898**	0.050	0.214	0.676
I-ATK				**1.000**	**0.998**	**0.996**	**1.000**	**1.000**	**0.998**
WI-ATK				0.694	0.782	**0.964**	0.624	0.704	**0.890**
I-PRK				**0.890**	0.770	**0.950**	**0.894**	0.686	**0.800**
WI-PRK				0.270	0.428	**0.924**	0.190	0.286	0.736
I-PSK				**0.918**	**0.906**	**0.858**	**0.926**	**0.934**	**0.896**
WI-PSK				0.322	0.492	**0.896**	0.280	0.390	0.724
				Concen-E-30%			Concen-E-70%		
DIF				0.092	0.362	**0.892**	0.062	0.244	**0.806**
I-ATK				**1.000**	**0.996**	**1.000**	**1.000**	**1.000**	**0.998**
WI-ATK				0.654	0.754	**0.972**	0.654	0.720	**0.928**
I-PRK				**0.880**	0.768	**0.958**	**0.864**	0.720	**0.866**
WI-PRK				0.254	0.422	**0.912**	0.222	0.312	0.756
I-PSK				**0.940**	**0.916**	**0.878**	**0.936**	**0.930**	**0.898**
WI-PSK				0.328	0.508	**0.904**	0.280	0.482	**0.812**

Note. Convergence rates greater than 0.8 were bolded. DIF = diffuse prior. I-ATK = informative prior at the true knot location. WI-ATK = weakly informative prior at the true knot location. I-PRK = informative prior at the pre-knot location. WI-PRK = weakly informative prior at the pre-knot location. I-PSK = informative prior at the post-knot location. WI-PSK = weakly informative prior at the post-knot location

### Convergence rate

Table 1 displays convergence rates for each simulation condition. Values in bold indicate convergence rates that are greater than 0.8. We begin with the complete data condition. When $n=50$, convergence rates were notably higher for informative priors (I-ATK, I-PRK, and I-PSK), with I-ATK showing the highest convergence rate. These high convergence rates were followed by weakly informative priors (WI-ATK, WI-PRK, and WI-PSK). Between the weakly informative priors, the convergence rate was considerably higher when the mean hyperparameter aligned with the true value (WI-ATK), in comparison with WI-PRK and WI-PSK. The lowest convergence rates were observed for DIF. These findings suggest that the use of priors with higher degrees of informativeness and better alignment with true values is advantageous for achieving higher convergence rates. When $n=150$, convergence rates increased across weakly informative and diffuse priors (DIF, WI-ATK, WI-PRK, and WI-PSK). This pattern highlights that, as sample sizes increase, the drawbacks of weakly or non-informative priors are mitigated and compensated with larger sample sizes. When $n=500$, all prior conditions achieved high convergence rates even greater than 0.9. It is evident that large sample sizes are advantageous with regard to obtaining high convergence.

As the proportion of missing data at the seventh time point increased to 30%, convergence rates decreased under the DIF condition, with a further noticeable decrease when 70% of the data were missing at the end. In contrast, convergence rates consistently remained (almost) 100% under the I-ATK setting, regardless of the sample sizes. These results highlight the advantages of using informative-accurate prior specifications in the presence of missing data, while the diffuse settings can suffer from low convergence rates. For the weakly informative prior settings (WI-ATK, WI-PRK, and WI-PSK), convergence rates tended to decrease under the 70% missing data conditions. While similar tendencies were observed for other prior settings, excluding I-ATK, the decreases were noticeable in the Consen-S-70% condition, even at the large sample size level. This pattern suggests that high proportions of missing data concentrated at the beginning can face challenges in achieving high convergence rates.

**Table 2:** Coverage rate

	*n* = 50	*n* = 150	*n* = 500	*n* = 50	*n* = 150	*n* = 500	*n* = 50	*n* = 150	*n* = 500
	Complete			Concen-S-30%			Consen-S-70%		
DIF	0.877	0.887	**0.927**	0.923	0.872	**0.924**	**0.936**	0.858	0.900
I-ATK	**0.996**	**0.990**	**0.968**	**1.000**	**0.994**	**0.974**	**1.000**	**0.994**	**0.990**
WI-ATK	**0.957**	**0.951**	**0.946**	**0.972**	**0.941**	**0.940**	**0.982**	**0.936**	**0.945**
I-PRK	0.006	0.062	0.396	0.008	0.044	0.333	0.002	0.030	0.167
WI-PRK	0.683	0.858	**0.923**	0.680	0.847	**0.921**	0.631	0.830	**0.901**
I-PSK	0.010	0.034	0.352	0.002	0.014	0.203	0.000	0.000	0.040
WI-PSK	0.614	0.749	0.880	0.571	0.656	0.879	0.471	0.488	0.736
				Constant-30%			Constant-70%		
DIF				0.884	0.849	**0.933**	**0.939**	0.858	**0.909**
I-ATK				**1.000**	**0.988**	**0.970**	**0.998**	**0.986**	**0.972**
WI-ATK				**0.972**	**0.933**	**0.942**	**0.984**	**0.931**	**0.937**
I-PRK				0.012	0.060	0.354	0.002	0.034	0.257
WI-PRK				0.695	0.866	**0.928**	0.613	0.806	**0.918**
I-PSK				0.002	0.008	0.242	0.000	0.002	0.082
WI-PSK				0.567	0.687	0.891	0.511	0.515	0.821
				Concen-E-30%			Concen-E-70%		
DIF				0.892	0.887	**0.935**	**0.922**	0.862	**0.916**
I-ATK				**1.000**	**1.000**	**0.968**	**0.998**	**0.996**	**0.972**
WI-ATK				**0.970**	**0.949**	**0.944**	**0.969**	**0.928**	**0.945**
I-PRK				0.006	0.056	0.357	0.000	0.028	0.336
WI-PRK				0.704	0.857	**0.926**	0.652	0.837	**0.932**
I-PSK				0.010	0.010	0.250	0.002	0.008	0.141
WI-PSK				0.611	0.698	0.864	0.566	0.605	0.825

Note. Coverage rates greater than 0.9 were bolded. DIF = diffuse prior. I-ATK = informative prior at the true knot location. WI-ATK = weakly informative prior at the true knot location. I-PRK = informative prior at the pre-knot location. WI-PRK = weakly informative prior at the pre-knot location. I-PSK = informative prior at the post-knot location. WI-PSK = weakly informative prior at the post-knot location

### Coverage rate

Table 2 presents coverage rates under different simulation conditions. We boldfaced values exceeding 0.9. When there were no missing data and the sample size was 50, we observed high coverage rates for I-ATK and WI-ATK, with I-ATK exhibiting the highest coverage rate. These results indicate the importance of alignment with the true value and higher informativeness for obtaining high coverage rates. Following WI-ATK, DIF showed high coverage rages, which were followed by WI-PRK and WI-PSK. The lowest coverage rates were observed under I-PRK and I-PSK, highlighting the negative effect of misaligned informative priors on coverage rates. As sample sizes increased to 150, low coverage rates under DIF or misaligned prior conditions (I-PRK, WI-PRK, I-PSK, and WI-PSK) increased, and further increases were observed when sample sizes amounted to 500. This demonstrates the role of larger sample sizes in achieving higher coverage rates.

In the presence of missing data, priors placed at the correct location (I-ATK and WI-ATK) were the least affected, maintaining high coverage rates with slight variations, regardless of the proportion of missing values at the final time point. When informative priors were placed at incorrect locations (I-PRK and I-PSK), coverage rates decreased further as the proportion of missing data increased to 30% and 70%. Such decreases were obvious for $n=150$ and $n=500$. Meanwhile, coverage rates remained relatively consistent or showed a slight decrease under the DIF condition when the proportion of missing data was 70% at the last time point. Particularly for $n=50$, coverage rates were higher in the DIF condition compared to conditions with priors placed at incorrect locations (I-PRK, WI-PRK, I-PSK, and WI-PSK).

**Fig. 4:**
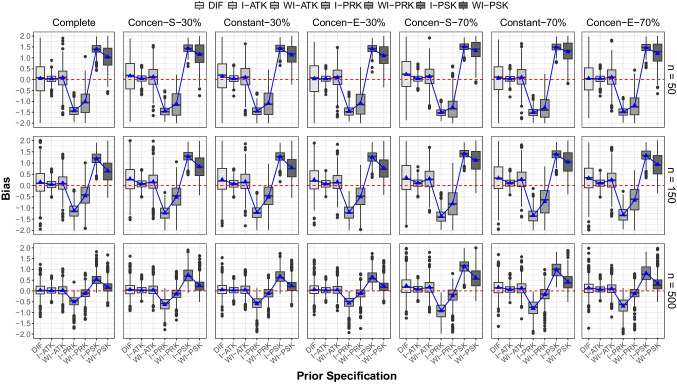
Boxplots of bias for knot location estimates across simulation conditions

### Bias

We present Fig. 4 to report bias across all simulation conditions. Rows in the figure correspond to different sample sizes, and columns represent each level of the missing data condition. The *x*-axis and *y*-axis denote prior specifications and the values of bias, respectively. The dashed horizontal line at 0 serves as a reference for no bias. To visualize average bias, triangular dots are plotted in each condition. However, due to some severely skewed or outlier parameter estimates, we additionally created boxplots to examine medians of bias across converged replication. Therefore, Fig. 4 not only displays average bias but also reveals the degree of deviations in parameter estimates from the true value at converged replications. Each of the triangular dots and boxplots corresponds to one of the seven prior specifications (from left to right: DIF, I-ATK, WI-ATK, I-PRK, WI-PRK, I-PSK, and WI-PSK) in respective simulation conditions.

When the data were complete and $n=50$, the bias was the most minimal for I-ATK, while the highest bias was found for informative priors with misaligned centers (I-PRK and I-PSK). It is clear that specifying informative priors with centers aligned to the true location leads to the lowest bias. Conversely, when informative priors are misplaced, parameter estimates can be biased to the largest extent. Using diffuse prior settings seems preferable to misaligned informative priors to avoid high bias, given that diffuse priors generally had little to no bias. In addition, for priors placed before the true location (I-PRK and WI-PRK), estimates were negatively biased, while for those placed after the true knot location (I-PSK and WI-PSK), estimates were positively biased. These findings highlight that locating prior distributions impacts the parameter estimation by shifting the direction of bias. As the sample size increased to 150 and 500, the degree of bias decreased. This indicates the effect of larger sample sizes in mitigating bias. For $n=500$, boxplots of WI-PRK and WI-PSK touched the dashed horizontal line, suggesting that bias with misaligned priors can be alleviated by incorporating weakly informative prior specifications.

When 30% of data were missing at the final time point, bias increased across different prior settings and sample size conditions because boxplots deviated from the dashed horizontal line. As the proportion of missing data increased to 70% at the last time point, we observed even greater degrees of bias across the simulation conditions. However, such increases in bias were minimal for I-ATK. In particular, we focus on the $n=50$ condition within the 70% missing data scenarios. When informative priors were accurately located (I-ATK), bias hardly increased. This demonstrates that informative and accurate priors can aid the negative consequences of missing data. Among the three attrition patterns, no substantial differences were detected.

**Table 3:** Root mean squared error

	*n* = 50	*n* = 150	*n* = 500	*n* = 50	*n* = 150	*n* = 500	*n* = 50	*n* = 150	*n* = 500
	Complete			Concen-S-30%			Consen-S-70%		
DIF	0.920	0.737	0.335	0.890	0.816	0.359	0.867	0.914	0.614
I-ATK	0.202	0.211	0.199	0.202	0.222	0.201	0.183	0.234	0.216
WI-ATK	0.553	0.462	0.275	0.541	0.529	0.302	0.540	0.597	0.434
I-PRK	1.458	1.191	0.580	1.478	1.272	0.706	1.525	1.417	1.004
WI-PRK	1.377	0.840	0.316	1.443	0.936	0.360	1.562	1.229	0.588
I-PSK	1.415	1.228	0.630	1.446	1.316	0.817	1.520	1.433	1.180
WI-PSK	1.326	0.883	0.349	1.396	1.092	0.434	1.609	1.338	0.839
				Constant-30%			Constant-70%		
DIF				0.920	0.781	0.336	0.940	0.919	0.453
I-ATK				0.207	0.223	0.196	0.179	0.229	0.212
WI-ATK				0.575	0.512	0.284	0.527	0.571	0.367
I-PRK				1.469	1.261	0.670	1.518	1.384	0.890
WI-PRK				1.378	0.909	0.317	1.548	1.203	0.499
I-PSK				1.442	1.307	0.754	1.505	1.403	1.038
WI-PSK				1.360	1.033	0.393	1.558	1.292	0.604
				Concen-E-30%			Concen-E-70%		
DIF				0.917	0.759	0.344	0.883	0.843	0.433
I-ATK				0.201	0.217	0.198	0.199	0.230	0.208
WI-ATK				0.548	0.502	0.288	0.529	0.535	0.338
I-PRK				1.476	1.258	0.642	1.498	1.351	0.797
WI-PRK				1.412	0.924	0.312	1.493	1.075	0.400
I-PSK				1.438	1.291	0.727	1.479	1.359	0.907
WI-PSK				1.377	1.031	0.391	1.484	1.180	0.542

Note. DIF = diffuse prior. I-ATK = informative prior at the true knot location. WI-ATK = weakly informative prior at the true knot location. I-PRK = informative prior at the pre-knot location. WI-PRK = weakly informative prior at the pre-knot location. I-PSK = informative prior at the post-knot location. WI-PSK = weakly informative prior at the post-knot location

### RMSE

Table 3 showcases RMSE across the simulation conditions considered. Overall, the observed patterns of RMSE aligned closely with those of bias. For this reason, we briefly point out the patterns recognized.

The smallest RMSE was attained when using informative priors at the true location (I-ATK). Conversely, the largest RMSE results were obtained with informative priors at incorrect locations (I-PRK and I-PSK), indicating that the misplacement of informative priors can lead to a greater dispersion of parameter estimates. In addition, we observed the influence of sample size: RMSE was the smallest for $n=500$ in each level of the missing data and prior conditions. Finally, as the amount of missing data increased, RMSE tended to increase, indicating the negative effects of missing data on the dispersion of parameter estimates.

**Fig. 5:**
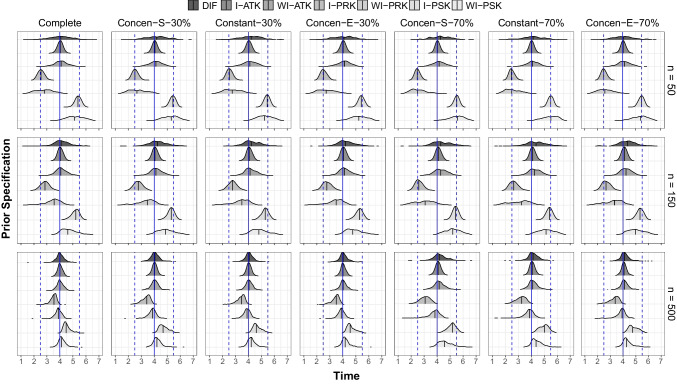
Distributions of posterior means for knot location across simulation conditions

### Posterior means of knot location

Figure 5 provides a collection of ridgeline plots displaying the distributions of posterior means for knot locations across simulation conditions. Each column corresponds to each level of the missing data condition, and rows represent three different sample sizes. Within each combination of missing data and sample size levels, seven respective distributions of posterior means for knot locations are described, each corresponding to different prior specifications. Moving from top to bottom, these distributions represent the posterior means for knot locations under the following prior distributions: DIF, I-ATK, WI-ATK, I-PRK, WI-PRK, I-PSK, and WI-PSK. Within each distribution, a vertical line marks the median to denote the center of the distribution.

We first examined conditions with complete data. When $n=50$, we observed that the centers of the distributions for DIF, I-ATK, and WI-ATK were located at the true location. The centers for I-PRK and WI-PRK were at or close to time point 2.5, and the centers for I-PSK and WI-PSK were at or close to time point 5.5. These patterns suggest that different prior specifications govern knot location estimates, particularly for small sample size conditions. In addition, we found that the informativeness of the prior settings influenced the dispersion of the distributions. For informative priors (I-ATK, I-PRK, and I-PSK), the dispersion was smaller and the knot location estimates gathered around their respective centers. For weakly informative priors (WI-ATK, WI-PRK, and WI-PSK), the estimates were more dispersed around their centers compared with informative priors. For diffuse priors, the posterior means scattered across all time points. When $n=150$, we noticed a shift in the centers for I-PRK, WI-PRK, I-PSK, and WI-PSK moving toward time point 4. This shift indicates that the impact of different prior specifications diminishes as sample sizes increase. As the sample size further increased to 500, the centers of all distributions were positioned at or near the true location. In particular, the shapes of distributions under DIF, I-ATK, and WI-ATK were quite similar. However, for informative priors placed at incorrect locations (I-PRK and I-PSK), the centers were farthest from the true location. These results highlight that knot locations were generally recovered around the true location under the largest sample size condition; yet, there existed the negative impact of informative and inaccurate priors on the estimation of knot locations.

As the proportion of missing data increased to 30% at the seventh time point, different patterns were found depending on sample size. For $n=50$, the centers for DIF, I-ATK, and WI-ATK remained at the true location. For the other prior settings, the centers were around time point 2.5 for I-PRK and WI-PRK, while for I-PSK and WI-PSK, the centers were around time point 5.5. When $n=150$, the centers for I-PRK, WI-PRK, I-PSK, and WI-PSK shifted toward the true location, and a further shift toward the true location was found for $n=500$. This observation means that, even in the presence of missing data, larger sample sizes wield more influence in determining the centers of the distributions for knot location estimates compared to prior distributions. When the proportion of missing data amounted to 70% at the end, the overall patterns were very similar to those under 30% of missing data conditions. However, the centers of the incorrectly placed priors (I-PRK, WI-PRK, I-PSK, and WI-PSK) were farther from the time point 4 compared to conditions under the 30% of missing at the end. In the 70% of missing data conditions, we could additionally observe that the centers of misplaced priors were the farthest under the Concen-S-70% condition.

**Fig. 6:**
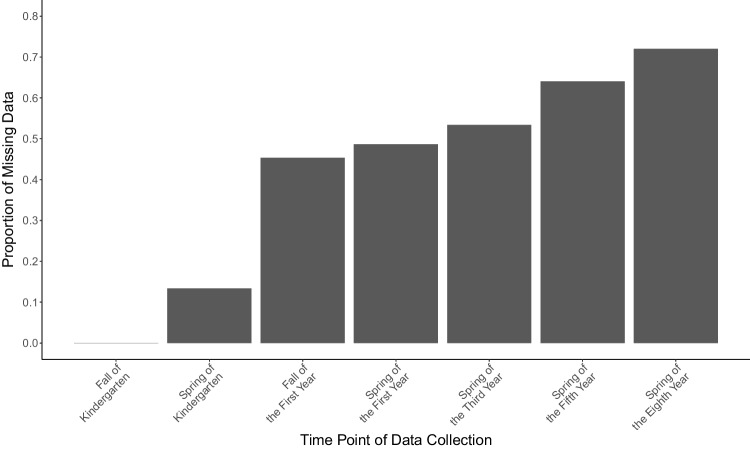
Attrition pattern for the ECLS-K data

## Illustrative example

We demonstrate the Bayesian estimation of knot locations in the presence of missing data characterized by attrition patterns, using a real dataset from the Early Childhood Longitudinal Studies - Kindergarten Cohort of 1998 (ECLS-K; National Center for Education Statistics - United States Department of Education, 2013). The ECLS-K study aimed to investigate various aspects of children’s early school experiences in a longitudinal framework. A particularly interesting aspect has been mathematics achievement, which is known to exhibit piecewise growth patterns, as indicated in methodological and applied studies (e.g., Kohli et al., 2015; Kohli & Sullivan, 2019; Marvin et al., 2023). These previous studies focused on piecewise patterns of mathematics achievements in the complete data scenarios. This illustrative example considers ECLS-K data with missing values and allows us to examine the estimation of changepoints in missing attrition scenarios under different prior settings. We provide the ECLS-K dataset, R code, and JAGS syntax used in the Illustrative Example section as online supplementary materials.

### Data

The ECLS-K data consisted of a nationally representative sample of approximately 21,000 children from the United States who began kindergarten in the 1998–1999 academic year. Mathematics achievement was measured by mathematics item response theory (IRT) scores across seven time points: the fall and spring of kindergarten, fall and spring of first grade, and the spring of third, fifth, and eighth grades. We coded these time points as 0, 0.5, 1, 1.5, 3.5, 5.5, and 8.5, respectively. To reflect a medium sample size and make our illustration applicable to realistic scenarios, we selected a random subsample of 150 children from the full dataset, who had complete data in the fall of kindergarten and either complete or missing responses from the spring of kindergarten through the spring of the eighth grade for math IRT scores. We made sure the missing data patterns from the selected children followed the attrition pattern. In particular, we found that the attrition pattern from the sample followed a decelerated increase in missing values, which was one of the missing data conditions in the simulation setup. At the final time point, 72% of the data were missing. Figure 6 illustrates this attrition pattern in our subsample from the ECLS-K data. The math IRT scores measured across seven time points were used as the outcome variable.

### Analysis

We estimated PGMs under seven different prior distributions. For illustrative purposes, we considered prior specifications similar to those utilized in the simulation design^3^. Specifically, we adjusted the hyperparameters of the uniform and truncated normal priors as follows:

- For DIF, $\gamma \sim \mathcal {U}(0, 8.5)$. This uniform distribution suggests that any values for knot locations from the first to the last time points, coded as 0 and 8.5, respectively, are equally likely.
- For I-ATK, $\gamma \sim \mathcal {N}(4.25, 0.417) \, \mathcal {T}(0, 8.5)$. The mean hyperparameter of 4.25 represents the midpoint of the time points, substantively the middle of the fall semester of the fourth grade. The variance hyperparameter of 0.417 is determined in a way that informative priors overlap with each other by 10%.
- For WI-ATK, $\gamma \sim \mathcal {N}(4.25, 2.085) \, \mathcal {T}(0, 8.5)$. The mean hyperparameter is the same as I-ATK. The variance hyperparameter of 2.085 is calculated by multiplying 5 by the variance of the informative prior distribution.
- For I-PRK, $\gamma \sim \mathcal {N}(2.125, 0.417) \, \mathcal {T}(0, 8.5)$. The mean hyperparameter is the center between the first time point and the middle of the fall semester of the fourth grade.
- For WI-PRK, $\gamma \sim \mathcal {N}(2.125, 2.085) \, \mathcal {T}(0, 8.5)$. This prior has the same mean hyperparameter as I-PRK but has a larger variance for the weakly informative setting.
- For I-PSK, $\gamma \sim \mathcal {N}(6.375, 0.417) \, \mathcal {T}(0, 8.5)$. The mean hyperparameter is centered between the midpoint of fourth grade and the last time point.
- For WI-PSK, $\gamma \sim \mathcal {N}(6.375, 2.085) \, \mathcal {T}(0, 8.5)$. This prior distribution uses the same mean hyperparameter as I-PSK with the variance hyperparameter value for the weakly informative prior.

Prior distributions for parameters other than knot locations were consistent with those used in the simulation design. With all the prior specifications set, we used the R rjags package for Bayesian analyses. We implemented four chains via Gibbs sampling, each chain consisting of 50,000 iterations for burn-in followed by 50,000 additional iterations for posterior inference, with a thinning interval of 1.

### Results

All models indicated satisfactory evidence of convergence with the $\widehat{R}$ statistics lower than 1.1. We report the summary of posterior distributions of knot locations in Table 4, which includes density plots, posterior means, posterior medians, posterior standard deviations, and 95% credible intervals under seven prior specifications. In the density plots, the blue solid line is the posterior mean, the green dashed line indicates the posterior median, and the two red dotted lines respectively mark the $2.5^{\text {th}}$ and $97.5^{\text {th}}$ percentiles in credible intervals.

For all prior conditions, the posterior means of knot locations suggested a changepoint during the fall of the fourth grade. The posterior means ranged from 4.176 to 4.368, indicating timing around the middle of the fall of the fourth year. The minimum and maximum values, 4.176 and 4.368, respectively, were observed under I-PRK and I-PSK. These results are understandable given that these two priors are informative, potentially pulling estimates toward a presumed location. However, there were minimal fluctuations in estimates across the conditions. Overall, this finding indicates that the changepoint in mathematics achievement likely occurs during the fall of the fourth grade.

**Table 4:** Summary of posterior distributions of knot locations from ECLS-K Data

	DIF	I-PRK	I-ATK	I-PSK
				
Mean	4.288	4.176	4.279	4.368
Median	4.292	4.182	4.284	4.368
SD	0.138	0.148	0.138	0.135
95% CI	[4.010, 4.550]	[3.861, 4.449]	[3.995, 4.538]	[4.102, 4.631]
		WI-PRK	WI-ATK	WI-PSK
				
Mean		4.263	4.282	4.305
Median		4.268	4.286	4.307
SD		0.138	0.142	0.140
95% CI		[3.980, 4.520]	[3.991, 4.552]	[4.021, 4.577]

Note. DIF = diffuse prior. I-ATK = informative prior at the true knot location. WI-ATK = weakly informative prior at the true knot location. I-PRK = informative prior at the pre-knot location. WI-PRK = weakly informative prior at the pre-knot location. I-PSK = informative prior at the post-knot location. WI-PSK = weakly informative prior at the post-knot location. SD = standard deviation. CI = credible interval

## Discussion

In Bayesian PGMs, the knot location is an important parameter because it indicates the time at which transitions occur between phases and denotes theoretically and substantively meaningful moments. While researchers can predetermine knot locations based on substantive theories or research design, a more flexible approach is to estimate knot locations using collected data. An important feature of Bayesian PGMs is that researchers can incorporate prior information to estimate knot locations. However, the estimation of knot locations can be affected by the presence of missing data that are ubiquitous in longitudinal studies. There is a research gap in exploring the impact of these two factors on recovering knot placements—an understanding critical for drawing accurate research conclusions about when to change. The objective of the current investigation was to examine the impact of prior specifications and missing data on the recovery of knot placements via a Monte Carlo simulation study and to demonstrate applications using illustrative examples. Next, we discuss key simulation results, provide advice for researchers, and suggest future research directions.

### Different prior specifications

We examined several prior specifications in the context of knot locations. By incorporating uniform and truncated normal distributions into the simulation setup, we manipulated the degree of informativeness and alignment with the true knot location. The evaluation of parameter recovery under outcome measures carried different nuances regarding the recovery of knot placement. In general, we observed the advantages of informative-accurate prior specifications on recovering true knot placement, which was especially evident in conditions with small sample sizes. These advantages encompassed high convergence and coverage rates, low bias and RMSE, and less dispersed distributions of knot location estimates around the true knot location. Given these advantages, we see informative and accurate priors as potential solutions to address the low convergence rates encountered by Kohli et al. (2015) when estimating Bayesian piecewise growth mixture models with small sample sizes. While researchers may consider increasing the number of iterations to improve convergence rates (Lock et al., 2018), the use of informative and accurate priors is also promising for achieving quality recovery.

In practice, where the true knot location is unknown or researchers have limited prior knowledge, specifying informative priors at wrong locations may result in low coverage rates and high bias and RMSE. In this regard, the use of informative priors should be approached cautiously. The benefits are contingent on accurate placement at the true location; on the other hand, misplacement may yield negative consequences. A reasonable alternative when partial knowledge is available is to consider specifying weakly informative priors around hypothesized knot locations. Such an alternative still offers benefits in terms of high coverage rates, low bias, and low RMSE, particularly with at least medium sample sizes.

The higher influence of prior distributions was diluted as sample sizes increased. This result is not surprising because Bayesian inference is the compromise between prior knowledge and obtained data (e.g., Gelman et al., 2013; van de Schoot et al., 2021). The decreasing influence of prior distributions with larger sample sizes was evident in the distributions of posterior means for knot locations. As sample sizes increased from 50 to 500, centers of distributions for misplaced prior distributions shifted toward the true location. Therefore, it is important to recognize the sample size effect in conjunction with the impact of different prior distributions. Obtaining higher sample sizes when available is recommended to mitigate the undesirable outcomes associated with using misplaced prior distributions in estimating knot locations.

### Presence of missing data

The simulation results revealed challenges in recovering knot placements due to the presence of missing data, which complicated the recovery associated with certain prior specifications. Increasing proportions of missing data resulted in negative consequences for parameter recovery, such as higher bias, particularly when priors were incorrectly located. These findings add new insights to the existing works (e.g., Graham, 2009; Nicholson et al., 2017; Shi et al., 2021), such that the use of informative and inaccurate priors hampers parameter recovery under missing data scenarios. Negative parameter recovery results, such as increased bias, were observed even with diffuse prior settings under large proportions of missing data. In addition, when priors were misplaced, the centers of distributions of posterior means shifted towards incorrect knot locations. We also observed interactions between the amount and pattern of missing data for certain outcome measures. In comparison with other simulation conditions, a large amount of missing data at the beginning was associated with noticeable decreases in convergence rates and a greater distance between the centers of the distributions of posterior mean estimates and the true knot location. This finding resonates with Heo et al. (2024) and emphasizes the negative consequences of a large amount of missingness in the beginning. Therefore, it is recommended that applied researchers carefully plan data collection strategies to minimize participant dropouts in the earlier stages of a study.

Our investigation uncovered that the use of informative or weakly informative priors placed at the true location could maintain high coverage rates and prevent further increases in bias in the presence of missing data. In addition, the centers of distributions of posterior mean estimates were consistently located at the true knot placement. Therefore, we emphasize that researchers can take advantage of Bayesian inference by leveraging available information when finding knot locations in the presence of missing data. In comparison with findings by Heo et al. (2024) that observed a negligible impact of informative and accurate priors on latent means in Bayesian model evaluation under missing data scenarios, our work adds to the field by demonstrating the sensitivity of knot locations to informative prior settings in accurately estimating knot locations.

### Advice for researchers

#### Which priors should we choose?

Researchers may wonder which priors should be chosen. It might seem reasonable to use diffuse priors as long as models converge, given the challenge of finding informative and accurate priors in practice. Such reasoning seems persuasive because our simulation results show that, when sample sizes are large, diffuse priors tend to yield parameter estimates closer to the true values. However, obtaining large samples is not always feasible, and what constitutes a “large” sample can vary by context. Diffuse priors also contribute to greater posterior uncertainty, leading to wider credible intervals and increased sensitivity in smaller samples. Another concern is that even researchers who make every effort to specify meaningful priors may inadvertently use informative yet inaccurate priors without realizing they are introducing bias. Indeed, our results indicate that, even with large sample sizes, incorrectly specified informative priors can lead to biased estimates, a problem exacerbated by missing data. One possible way to mitigate the strong influence of an inaccurate prior is to increase its variance (Depaoli, 2014). Still, all prior distributions inevitably contain some level of inaccuracy that can substantially influence the estimation of knot locations.

Our advice is to include prior sensitivity analyses as an integral part of analysis plans for a confident and thorough examination (Depaoli et al., 2020; van de Schoot et al., 2018). Conducting these analyses helps researchers avoid relying on a single prior: whether it is an informative yet inaccurate prior that might be misleading or a diffuse prior that can introduce unnecessary uncertainty. Prior sensitivity analyses allow researchers to evaluate how final estimates respond to different priors and to detect any discrepancies in the outcomes. For a more comprehensive understanding of prior choices, it is usually recommended to compare the impact of different priors, ranging from diffuse to weakly informative or informative (Depaoli et al., 2017; Depaoli & van de Schoot, 2017). For transparency, researchers should report results under each prior specification. If the results from sensitivity analyses fluctuate under different prior specification scenarios, it signals that the data may not align with the substantive theories on changepoints. Then, researchers may consider the need for follow-up studies under different conditions to test the theories (e.g., different sample characteristics, smaller or larger sample sizes, or alternative prior specifications). However, if the results are robust and estimates show minimal fluctuations, as shown in our illustrative examples, it suggests that the results are stable against the supposed inaccuracy, and the underlying theory is strong and reinforces confidence in the knot location estimates.

We acknowledge that specifying prior distributions in a proper and accurate manner is not always intuitive. Researchers can derive priors based on previous findings or meta-analysis (see Heo et al., 2024, for examples of specifying priors in mixture PGMs using earlier study results). However, available prior knowledge may still be limited by existing literature. As an alternative, researchers can consider prior elicitation. Through eliciting expert knowledge, information or insight from experts is aggregated and extracted to construct probabilistic representations of this knowledge, which are employed as prior distributions. This way, experts’ prior knowledge is incorporated into the analysis. For guidance on eliciting priors based on expert judgment, see Veen et al. (2017). Specifically for latent growth modeling, see van de Schoot et al. (2018) and Veen et al. (2020). In small-sample settings, Zondervan-Zwijnenburg et al. (2017) offer useful strategies. For a broader overview of eliciting prior knowledge, Mikkola et al. (2024) is a comprehensive resource.

#### How can we prevent missing data?

Researchers can take several proactive actions to prevent or minimize the occurrence of missing data or attrition, such as clear communication and the use of refined data-collection instruments. In addition, in the design phase of a longitudinal study, researchers have the option to implement planned missing data designs as data collection strategies (Rhemtulla et al., 2014; Wu & Jia, 2021). In planned missing data designs, participants are randomly assigned to respond at a subset of measurement occasions to help reduce participant fatigue and potentially lower attrition rates. Because planned missing data are MCAR, parameter estimates remain unbiased. In addition, planned missing data designs offer advantages in terms of higher efficiency by achieving a better balance between the number of repeated measures for each participant and the overall sample size (Wu et al., 2016). This contributes to a reduced budget and allows for the collection of a larger number of participants, which can increase statistical power.

### Practical challenges and future directions

The current work lays the foundation for future research aimed at explaining and addressing the issues surrounding knot locations. We used a linear-linear PGM with one unknown changepoint as the population model to systematically evaluate the interaction between priors and missing data, making this study one of the first investigations into missing data and parameter recovery issues in Bayesian PGMs. However, PGMs can be extended into more complex formats, including models with multiple knot locations, mixture components, and individually varying knot locations (e.g., Harring et al., 2021; Heo et al., 2024; Kohli et al., 2015; Lock et al., 2018; Peralta et al., 2022). These extensions introduce several practical challenges that warrant further investigation.

For instance, increasing the number of knots expands the parameter space. In such settings, the computational burden increases, and the use of diffuse priors could lead to convergence difficulties or unreliable estimation, particularly in small sample conditions (Kohli et al., 2015). One possible approach is to employ weakly informative priors if substantive knowledge suggests likely intervals where knot shifts typically occur. Here, each knot location requires its own distribution, and constraints on hyperparameters can be considered (Lock et al., 2018). Nevertheless, follow-up research is needed to develop computationally efficient yet flexible prior strategies that can reflect substantive theories and allow for sensitivity assessments in estimating multiple knot locations (see Heo et al., 2024, for examples of prior specification strategies and sensitivity analyses in mixture PGMs). When knots vary across latent classes, additional modeling challenges arise, such as label-switching issues, which can be handled by imposing parameter constraints or by using relabeling algorithms (Cassiday et al., 2021; Papastamoulis, 2016). Conceptually, the prior specification strategies considered in this article could be extended by assigning separate priors to different classes, although the performance of such approaches requires empirical validation.

The presence of missing data adds another layer of complexity when estimating knot locations in more complex PGMs. In the mixture modeling framework, missing data patterns might differ systematically across latent classes, making proper treatment of these distinct patterns crucial to avoid misclassification. When the true growth process features multiple changepoints, missing data patterns – such as whether missing values are concentrated at particular measurement occasions (Heo et al., 2024) – could distort the recovery of knot placements. In PGMs with individually varying knots, missing data may disproportionately affect certain individuals or subgroups. The current understanding of knot location estimation in the presence of missing data is still in its early stages. More comprehensive explorations of the interactions between complex PGMs and missing data deserve further attention in future research.

In addition, we focused on MAR data. Although MAR is a common assumption in methodological research, we note that true missing data mechanisms are usually unknown. Missingness can be nonignorable (Enders, 2011), and we recognize a possibility of a combination of different missing data mechanisms as pointed out in Graham (2009); Nicholson et al. (2017). The recovery of parameters in PGMs, including knot locations, in these complex missing data scenarios is still unknown. Future research should delve into these contexts to ensure unbiased parameter estimates.

In our simulation design, we generated data from seven equally spaced measurement occasions, with the knot located at the midpoint of the time period. However, the number of time points and the placement of knots away from the midpoint can play important roles in knot location estimation. For instance, having more measurement occasions generally provides more information for trajectory estimation. Meanwhile, suppose a knot is placed early in the measurement timeline. In that case, fewer observations are available to adequately anchor the pre-knot segment, hence complicating the knot location estimate, particularly if dropout occurs in those earlier waves. Although these aspects were not considered in our main simulation, we conducted a secondary simulation study to gain preliminary insight into knot location recovery when a knot is placed early, in the middle, or later among nine equally spaced measurements under one missing data scenario. While the secondary simulation study indicated that having more time points generally led to higher convergence rates and improved parameter recovery (e.g., lower bias, lower RMSE, higher coverage) as sample size increased, missing values still had an effect. Specifically, when the knot location was placed earlier, estimates tended to be underestimated, whereas placing the knot later led to overestimation. Desirable parameter recovery was observed when knot locations were accurately specified, but pronounced bias occurred when priors were incorrectly placed and farther from the true knot location. In the incorrect prior conditions that are the farthest, even the largest sample size did not fully mitigate this bias. To conserve space, we refer readers to the online supplementary materials for the full results^4^. Further investigation is needed to explore these scenarios under more complex conditions and missing data patterns.

### Conclusion

Our study has illustrated that research conclusions can vary depending on the specification of prior distributions and the nature of missing data. We reiterate that understanding the impact of these two factors is pivotal for reaching pronounced conclusions regarding changepoints. We are hopeful that our investigation will spark further explorations into modeling the timing of change and benefit methodologists and applied researchers interested in studying piecewise growth curves.

## Open Practices Statements

To enhance the accessibility and usability of our model and methods in this study, the datasets, R code, and JAGS syntax for both the simulations and the illustrative example are freely available at https://osf.io/j46bf/.

## Data Availability

The link to the data is provided in the References. The link to the materials is provided in the Open Practices Statements.

## Appendix A. Assessing convergence using different cutoff criteria

In the Bayesian piecewise growth modeling literature, different studies have used various cutoff criteria for assessing chain convergence. Peralta et al. (2022); Lock et al. (2018) used $\widehat{R} < 1.2$ as a cutoff, while Depaoli et al. (2023); Heo et al. (2024) used $\widehat{R}$ < 1.05. To analyze the impact of different cutoff criteria, we conducted a sensitivity analysis on convergence. Tables 5 and 6 present convergence rates for the two respective cutoff values (i.e., $\widehat{R}$ < 1.05 and $\widehat{R}$ < 1.2). When $\widehat{R}$ < 1.05 was used, the mean percentage of converged replications was 61.57%, with a median of 69.40%. With $\widehat{R}$ < 1.2, the mean percentage of converged replications was 80.36%, with a median of 92.40%. When more liberal criteria were used ($\widehat{R}$ < 1.2), the convergence rates increased substantially. These results highlight that different cutoff criteria have an impact on convergence rates. Other criteria, such as Monte Carlo standard errors on the order of 0.001 (Kohli et al., 2015) and Geweke statistics (Wang & McArdle, 2008), have been also used in evaluating chain convergence. This paper, however, used $\widehat{R}$ statistics. Thus, we did not evaluate convergence using Monte Carlo standard errors or Geweke statistics under different criteria.

**Table 5:** Convergence rate for $\widehat{R}$ < 1.05

	*n* = 50	*n* = 150	*n* = 500	*n* = 50	*n* = 150	*n* = 500	*n* = 50	*n* = 150	n=500
	Complete			Concen-S-30%			Consen-S-70%		
DIF	0.096	0.334	**0.928**	0.062	0.264	**0.816**	0.022	0.118	0.412
I-ATK	**0.998**	**0.982**	**1.000**	**0.998**	**0.984**	**0.998**	**1.000**	**0.992**	**0.976**
WI-ATK	0.496	0.628	**0.970**	0.486	0.606	**0.930**	0.454	0.584	0.716
I-PRK	0.744	0.592	**0.950**	0.742	0.530	**0.894**	0.754	0.442	0.656
WI-PRK	0.152	0.400	**0.938**	0.116	0.328	**0.880**	0.084	0.184	0.558
I-PSK	**0.846**	0.732	**0.832**	**0.846**	0.778	0.688	**0.864**	**0.844**	0.638
WI-PSK	0.242	0.420	**0.914**	0.184	0.346	0.784	0.158	0.248	0.452
				Constant-30%			Constant-70%		
DIF				0.064	0.258	**0.856**	0.026	0.154	0.566
I-ATK				**0.996**	**0.980**	**0.996**	**1.000**	**0.996**	**0.988**
WI-ATK				0.532	0.650	**0.948**	0.454	0.588	0.794
I-PRK				0.740	0.536	**0.926**	0.782	0.446	0.718
WI-PRK				0.140	0.340	**0.908**	0.088	0.206	0.692
I-PSK				**0.844**	0.774	0.750	**0.888**	**0.880**	0.694
WI-PSK				0.188	0.366	**0.838**	0.136	0.266	0.586
				Concen-E-30%			Concen-E-70%		
DIF				0.056	0.272	**0.860**	0.042	0.196	0.722
I-ATK				**0.996**	**0.980**	**0.998**	**1.000**	**1.000**	**0.992**
WI-ATK				0.494	0.604	**0.950**	0.468	0.616	**0.856**
I-PRK				0.778	0.536	**0.934**	0.730	0.460	0.794
WI-PRK				0.140	0.342	**0.898**	0.094	0.238	0.726
I-PSK				**0.854**	0.776	0.790	**0.880**	**0.880**	0.710
WI-PSK				0.192	0.394	**0.846**	0.160	0.380	0.690

Note. Convergence rates greater than 0.8 were bolded. DIF = diffuse prior. I-ATK = informative prior at the true knot location. WI-ATK = weakly informative prior at the true knot location. I-PRK = informative prior at the pre-knot location. WI-PRK = weakly informative prior at the pre-knot location. I-PSK = informative prior at the post-knot location. WI-PSK = weakly informative prior at the post-knot location

**Table 6:** Convergence Rate for $\widehat{R}$ < 1.2

	*n* = 50	*n* = 150	*n* = 500	*n* = 50	*n* = 150	*n* = 500	*n* = 50	*n* = 150	*n* = 500
	Complete			Concen-S-30%			Consen-S-70%		
DIF	0.262	0.544	**0.976**	0.230	0.460	**0.914**	0.164	0.300	0.636
I-ATK	**1.000**	**1.000**	**1.000**	**1.000**	**1.000**	**1.000**	**1.000**	**1.000**	**1.000**
WI-ATK	**0.822**	**0.884**	**0.990**	**0.822**	**0.866**	**0.984**	**0.834**	**0.836**	**0.930**
I-PRK	**0.964**	**0.930**	**0.984**	**0.938**	**0.924**	**0.964**	**0.944**	**0.894**	**0.854**
WI-PRK	0.504	0.638	**0.960**	0.514	0.582	**0.918**	0.452	0.480	0.700
I-PSK	**0.968**	**0.982**	**0.958**	**0.970**	**0.974**	**0.946**	**0.974**	**0.978**	**0.968**
WI-PSK	0.556	0.738	**0.962**	0.554	0.688	**0.932**	0.524	0.554	0.784
				Constant-30%			Constant-70%		
DIF				0.216	0.446	**0.940**	0.176	0.320	0.784
I-ATK				**1.000**	**1.000**	**1.000**	**1.000**	**1.000**	**1.000**
WI-ATK				**0.836**	**0.896**	**0.982**	**0.840**	**0.816**	**0.958**
I-PRK				**0.962**	**0.924**	**0.970**	**0.940**	**0.920**	**0.912**
WI-PRK				0.522	0.574	**0.936**	0.426	0.488	0.780
I-PSK				**0.964**	**0.976**	**0.946**	**0.966**	**0.982**	**0.978**
WI-PSK				0.550	0.676	**0.950**	0.492	0.638	**0.862**
				Concen-E-30%			Concen-E-70%		
DIF				0.230	0.484	**0.946**	0.174	0.368	**0.856**
I-ATK				**1.000**	**1.000**	**1.000**	**1.000**	**1.000**	**1.000**
WI-ATK				**0.830**	**0.876**	**0.984**	**0.824**	**0.850**	**0.980**
I-PRK				**0.940**	**0.928**	**0.990**	**0.942**	**0.902**	**0.928**
WI-PRK				0.502	0.588	**0.926**	0.478	0.526	0.786
I-PSK				**0.980**	**0.968**	**0.964**	**0.970**	**0.970**	**0.992**
WI-PSK				0.564	0.702	**0.946**	0.512	0.688	**0.884**

Note. Convergence rates greater than 0.8 were bolded. DIF = diffuse prior. I-ATK = informative prior at the true knot location. WI-ATK = weakly informative prior at the true knot location. I-PRK = informative prior at the pre-knot location. WI-PRK = weakly informative prior at the pre-knot location. I-PSK = informative prior at the post-knot location. WI-PSK = weakly informative prior at the post-knot location

## Footnotes

1 For the sake of coherence and simplicity, it is assumed that unless otherwise stated, piecewise growth modeling refers to piecewise growth curve modeling that has neither mixture nor mixed-effects components.

2 While this cutoff value is common in methodological research, we found that different cutoff values have been used in Bayesian piecewise modeling literature for assessing convergence. In light of this, we additionally conducted a sensitivity analysis of convergence rates under different cutoff values of $\widehat{R}$ statistics and reviewed convergence criteria in Appendix A.

3 The prior specifications in this illustrative example are chosen without relying on prior knowledge. While these specifications mimic realistic scenarios in Bayesian analysis by varying prior accuracy and informativeness, they are purely illustrative. In practice, researchers should derive their prior distributions from substantive theories or previous studies to make sure that the priors are appropriately accurate and informative, making substantive sense in the specific research context.

4 The full results, data, R code, and JAGS syntax for this secondary simulation study can be accessed as online supplementary materials.

## Availability of data and materials

The link to the code is provided in the Open Practices Statements.

## References

[CR1] Agresti, A. (2012). *Categorical data analysis* (3rd ed.). Hoboken: Wiley.

[CR2] Bollen, K. A., & Curran, P. J. (2006). *Latent curve models: A structural equation perspective*. Hoboken: John Wiley & Sons.

[CR3] Can, S., van de Schoot, R., & Hox, J. (2015). Collinear latent variables in multilevel confirmatory factor analysis: A comparison of maximum likelihood and bayesian estimations. *Educational and Psychological Measurement,**75*(3), 406–427. 10.1177/0013164414547959 29795827 10.1177/0013164414547959 PMC5965642

[CR4] Cassiday, K. R., Cho, Y., & Harring, J. R. (2021). A comparison of label switching algorithms in the context of growth mixture models. *Educational and Psychological Measurement,**81*(4), 668–697. 10.1177/0013164420970614 34267396 10.1177/0013164420970614 PMC8243206

[CR5] Chung, J. M., Hutteman, R., van Aken, M. A., & Denissen, J. J. (2017). High, low, and in between: Self-esteem development from middle childhood to young adulthood. *Journal of Research in Personality,**70*, 122–133. 10.1016/j.jrp.2017.07.001

[CR6] Cohen, J. (2002). *Statistical power analysis for the behavioral sciences*. London: Routledge.

[CR7] Cudeck, R., & Codd, C. L. (2012). A template for describing individual differences in longitudinal data, with application to the connection between learning and ability. In J. R. Harring & G. R. Hancock (Eds.), Advances in longitudinal methods in the social and behavioral sciences (pp. 3-24).

[CR8] Daniels, M. J., & Hogan, J. W. (2008). *Missing data in longitudinal studies: Strategies for Bayesian modeling and sensitivity analysis*. Chapman; hall/CRC.

[CR9] Depaoli, S. (2013). Mixture class recovery in gmm under varying degrees of class separation: Frequentist versus Bayesian estimation. *Psychological Methods,**18*(2), 186–219. 10.1037/a0031609 23527607 10.1037/a0031609

[CR10] Depaoli, S. (2014). The impact of inaccurate “informative’’ priors for growth parameters in Bayesian growth mixture modeling. *Structural Equation Modeling: A Multidisciplinary Journal,**21*(2), 239–252. 10.1080/10705511.2014.882686

[CR11] Depaoli, S., & Boyajian, J. (2014). Linear and nonlinear growth models: Describing a Bayesian perspective. *Journal of Consulting and Clinical Psychology,**82*(5), 784–802. 10.1037/a0035147 24364797 10.1037/a0035147

[CR12] Depaoli, S., Jia, F., & Heo, I. (2023). Detecting model misspecifications in Bayesian piecewise growth models. *Structural Equation Modeling: A Multidisciplinary Journal,**30*(4), 574–591. 10.1080/10705511.2022.2144865

[CR13] Depaoli, S., & van de Schoot, R. (2017). Improving transparency and replication in Bayesian statistics: The wambs-checklist. *Psychological Methods,**22*(2), 240. 10.1037/met0000065 26690773 10.1037/met0000065

[CR14] Depaoli, S., Winter, S. D., & Visser, M. (2020). The importance of prior sensitivity analysis in Bayesian statistics: Demonstrations using an interactive shiny app. *Frontiers in Psychology,**11*, Article 608045. 10.3389/fpsyg.2020.608045 33324306 10.3389/fpsyg.2020.608045 PMC7721677

[CR15] Depaoli, S., Yang, Y., & Felt, J. (2017). Using Bayesian statistics to model uncertainty in mixture models: A sensitivity analysis of priors. *Structural Equation Modeling: A Multidisciplinary Journal,**24*(2), 198–215. 10.1080/10705511.2016.1250640

[CR16] Diallo, T. M. O., Morin, A. J. S., & Parker, P. D. (2013). Statistical power of latent growth curve models to detect quadratic growth. *Behavior Research Methods,**46*, 357–371. 10.3758/s13428-013-0395-1 10.3758/s13428-013-0395-1 24234337

[CR17] Enders, C. K. (2011). Missing not at random models for latent growth curve analyses. *Psychological Methods,**16*(1), 1–16. 10.1016/j.jmp.2008.03.002 21381816 10.1037/a0022640

[CR18] Enders, C. K., & Mansolf, M. (2018). Assessing the fit of structural equation models with multiply imputed data. *Psychological Methods,**23*(1), 76–93. 10.1037/met0000102 27893216 10.1037/met0000102

[CR19] Flora, D. B. (2008). Specifying piecewise latent trajectory models for longitudinal data. *Structural Equation Modeling: A Multidisciplinary Journal,**15*(3), 513–533. 10.1080/10705510802154349

[CR20] Gamerman, D., & Lopes, H. F. (2006). *Markov chain monte carlo: Stochastic simulation for bayesian inference*. Boca Raton: CRC Press.

[CR21] Gelman, A., Carlin, J. B., Stern, H. S., Dunson, D. B., Vehtari, A., & Rubin, D. B. (2013). *Bayesian data analysis (3rd)*. FL: CRC Press Boca Raton.

[CR22] Geman, S., & Geman, D. (1984). Stochastic relaxation, gibbs distributions, and the bayesian restoration of images. *IEEE Transactions on Pattern Analysis and Machine Intelligence,**6*, 721–741. 10.1109/TPAMI.1984.4767596 22499653 10.1109/tpami.1984.4767596

[CR23] Graham, J. W. (2009). Missing data analysis: Making it work in the real world. *Annual Review of Psychology,**60*, 549–576. 10.1146/annurev.psych.58.110405.085530 18652544 10.1146/annurev.psych.58.110405.085530

[CR24] Grimm, K. J., Ram, N., & Estabrook, R. (2016). *Growth modeling: Structural equation and multilevel modeling approaches*. New York: Guilford Publications.

[CR25] Gustavson, K., von Soest, T., Karevold, E., & Røysamb, E. (2012). Attrition and generalizability in longitudinal studies: Findings from a 15-year population-based study and a monte carlo simulation study. *BMC Public Health,**12*(1), 1–11. 10.1186/1471-2458-12-918 23107281 10.1186/1471-2458-12-918 PMC3503744

[CR26] Hardy, S. A., & Thiels, C. (2009). Using latent growth curve modeling in clinical treatment research: An example comparing guided self-change and cognitive behavioral therapy treatments for bulimia nervosa. *International Journal of Clinical and Health Psychology,**9*(1), 51–71.

[CR27] Harring, J. R., Cudeck, R., & Du Toit, S. H. (2006). Fitting partially nonlinear random coefficient models as sems. *Multivariate Behavioral Research,**41*(4), 579–596. 10.1207/s15327906mbr4104_7 26794919 10.1207/s15327906mbr4104_7

[CR28] Harring, J. R., Strazzeri, M. M., & Blozis, S. A. (2021). Piecewise latent growth models: Beyond modeling linear-linear processes. *Behavior Research Methods,**53*(2), 593–608. 10.3758/s13428-020-01420-5 32779105 10.3758/s13428-020-01420-5

[CR29] Heo, I., Depaoli, S., Jia, F., & Liu, H. (2024). Bayesian approach to piecewise growth mixture modeling: Issues and applications in school psychology. *Journal of School Psychology,**107*, Article 101366. 10.1016/j.jsp.2024.101366 39645322 10.1016/j.jsp.2024.101366

[CR30] Heo, I., Jia, F., & Depaoli, S. (2024). Performance of model fit and selection indices for Bayesian piecewise growth modeling with missing data. *Structural Equation Modeling: A Multidisciplinary Journal,**31*(3), 455–476. 10.1080/10705511.2023.2264514

[CR31] Hu, B. Y., Fan, X., Wu, Y., LoCasale-Crouch, J., & Song, Z. (2020). Teacher-child interaction quality and Chinese children’s academic and cognitive development: New perspectives from piecewise growth modeling. *Early Childhood Research Quarterly,**51*, 242–255. 10.1016/j.ecresq.2019.10.003

[CR32] Jaggars, S. S., & Xu, D. (2016). Examining the earnings trajectories of community college students using a piecewise growth curve modeling approach. *Journal of Research on Educational Effectiveness,**9*(3), 445–471. 10.1080/19345747.2015.1116033

[CR33] Kohli, N., & Harring, J. R. (2013). Modeling growth in latent variables using a piecewise function. *Multivariate Behavioral Research,**48*(3), 370–397. 10.1080/00273171.2013.778191 26741847 10.1080/00273171.2013.778191

[CR34] Kohli, N., Hughes, J., Wang, C., Zopluoglu, C., & Davison, M. L. (2015). Fitting a linear-linear piecewise growth mixture model with unknown knots: A comparison of two common approaches to inference. *Psychological Methods,**20*(2), 259–275. 10.1037/met0000034 25867487 10.1037/met0000034

[CR35] Kohli, N., & Sullivan, A. L. (2019). Linear-linear piecewise growth mixture models with unknown random knots: A primer for school psychology. *Journal of school psychology,**73*, 89–100. 10.1016/j.jsp.2019.03.004 30961883 10.1016/j.jsp.2019.03.004

[CR36] Kristman, V., Manno, M., & Côté, P. (2004). Loss to follow-up in cohort studies: How much is too much? *European Journal of Epidemiology,**19*(8), 751–760. 10.1023/B:EJEP.0000036568.02655.f8 15469032 10.1023/b:ejep.0000036568.02655.f8

[CR37] Kroese, F. M., Adriaanse, M. A., Vinkers, C. D., van de Schoot, R., & de Ridder, D. T. (2014). The effectiveness of a proactive coping intervention targeting self-management in diabetes patients. *Psychology & Health,**29*(1), 110–125. 10.1080/08870446.2013.841911 10.1080/08870446.2013.841911 24111623

[CR38] Kwok, O.-M., Luo, W., & West, S. G. (2010). Using modification indexes to detect turning points in longitudinal data: A Monte Carlo study. *Structural Equation Modeling: A Multidisciplinary Journal,**17*(2), 216–240. 10.1080/10705511003659359

[CR39] Lee, I. H., & Rojewski, J. W. (2009). Development of occupational aspiration prestige: A piecewise latent growth model of selected influences. *Journal of Vocational Behavior,**75*(1), 82–90. 10.1016/j.jvb.2009.03.006

[CR40] Lee, S.-Y. (2007). *Structural equation modeling: A Bayesian approach*. Hoboken: John Wiley & Sons.

[CR41] Lee, S.-Y., & Song, X.-Y. (2004). Bayesian model comparison of nonlinear structural equation models with missing continuous and ordinal categorical data. *British Journal of Mathematical and Statistical Psychology,**57*(1), 131–150. 10.1348/000711004849204 15171804 10.1348/000711004849204

[CR42] Lee, S.-Y., & Tang, N.-S. (2006). Bayesian analysis of nonlinear structural equation models with nonignorable missing data. *Psychometrika,**71*(3), 541–564. 10.1007/s11336-006-1177-1

[CR43] Leite, W. L., & Stapleton, L. M. (2011). Detecting growth shape misspecifications in latent growth models: An evaluation of fit indexes. *The Journal of Experimental Education,**79*(4), 361–381. 10.1080/00220973.2010.509369

[CR44] Li, F., Duncan, T. E., & Hops, H. (2001). Examining developmental trajectories in adolescent alcohol use using piecewise growth mixture modeling analysis. *Journal of Studies on Alcohol*, *62*(2), 199-210. 10.15288/jsa.2001.62.199 10.15288/jsa.2001.62.199 11327186

[CR45] Little, R. J., & Rubin, D. B. (2002). *Statistical analysis with missing data*. Hoboken: John Wiley & Sons.

[CR46] Lock, E. F., Kohli, N., & Bose, M. (2018). Detecting multiple random changepoints in Bayesian piecewise growth mixture models. *Psychometrika,**83*(3), 733–750. 10.1007/s11336-017-9594-5 29150814 10.1007/s11336-017-9594-5 PMC6019237

[CR47] Marcoulides, K. M. (2018). Automated latent growth curve model fitting: A segmentation and knot selection approach. *Structural Equation Modeling: A Multidisciplinary Journal,**25*(5), 687–699. 10.1080/10705511.2018.1424548

[CR48] Marvin, L., Liu, H., & Depaoli, S. (2023). Using Bayesian piecewise growth curve models to handle complex nonlinear trajectories. *Journal of Behavioral Data Science*, *3*(1), 1-33. 10.35566/jbds/v3n1/marvin

[CR49] McNeish, D. M. (2016). On using Bayesian methods to address small sample problems. *Structural Equation Modeling: A Multidisciplinary Journal,**23*(5), 750–773. 10.1080/10705511.2016.1186549

[CR50] McNeish, D. M. (2016). Using data-dependent priors to mitigate small sample bias in latent growth models: A discussion and illustration using m plus. *Journal of Educational and Behavioral Statistics,**41*(1), 27–56. 10.3102/1076998615621299

[CR51] Meredith, W., & Tisak, J. (1990). Latent curve analysis. *Psychometrika,**55*, 107–122. 10.1007/BF02294746

[CR52] Mikkola, P., Martin, O. A., Chandramouli, S., Hartmann, M., Abril Pla, O., Thomas, O., Pesonen, H., Corander, J., Vehtari, A., Kaski, S., et al. (2024). Prior knowledge elicitation: The past, present, and future. *Bayesian Analysis,**19*(4), 1129–1161. 10.1214/23-BA1381

[CR53] National Center for Education Statistics - United States Department of Education. (2013). Early Childhood Longitudinal Study [United States]: Kindergarten Class of 1998-1999 [Inter-university Consortium for Political and Social Research [distributor]]. 10.3886/ICPSR03676.v1

[CR54] Nicholson, J. S., Deboeck, P. R., & Howard, W. (2017). Attrition in developmental psychology: A review of modern missing data reporting and practices. *International Journal of Behavioral Development,**41*(1), 143–153. 10.1177/0165025415618275

[CR55] Ning, L., & Luo, W. (2017). Specifying turning point in piecewise growth curve models: Challenges and solutions. *Frontiers in Applied Mathematics and Statistics,**3*, 19. 10.3389/fams.2017.00019

[CR56] Ortega-Azurduy, S., Tan, F., & Berger, M. (2008). The effect of dropout on the efficiency of d-optimal designs of linear mixed models. *Statistics in Medicine,**27*(14), 2601–2617. 10.1002/sim.3108 17943923 10.1002/sim.3108

[CR57] Papastamoulis, P. (2016). Label.switching: An R package for dealing with the label switching problem in MCMC outputs. *Journal of Statistical Software*, *69*(1), 1-24. 10.18637/jss.v069.c01

[CR58] Patrick, M. E., & Schulenberg, J. E. (2011). How trajectories of reasons for alcohol use relate to trajectories of binge drinking: National panel data spanning late adolescence to early adulthood. *Developmental Psychology,**47*(2), 311–317. 10.1037/a0021939 21219061 10.1037/a0021939 PMC3058882

[CR59] Peralta, Y., Kohli, N., Lock, E. F., & Davison, M. L. (2022). Bayesian modeling of associations in bivariate piecewise linear mixed-effects models. *Psychological Methods,**27*(1), 44–64. 10.1037/met0000358 33030911 10.1037/met0000358

[CR60] Plummer, M., et al. (2003). Jags: A program for analysis of Bayesian graphical models using Gibbs sampling. *Proceedings of the 3rd international workshop on distributed statistical computing*, *124*(125.10), 1-10.

[CR61] Plummer, M. (2016). Rjags: Bayesian graphical models using mcmc. https://cran.r-project.org/web/packages/rjags/index.html

[CR62] Raudenbush, S. W., & Liu, X.-F. (2001). Effects of study duration, frequency of observation, and sample size on power in studies of group differences in polynomial change. *Psychological Methods,**6*(4), 387–401. 10.1037/1082-989X.6.4.387 11778679

[CR63] Rhemtulla, M., Jia, F., Wu, W., & Little, T. D. (2014). Planned missing designs to optimize the efficiency of latent growth parameter estimates. *International Journal of Behavioral Development,**38*(5), 423–434. 10.1177/0165025413514324

[CR64] Robert, C., & Casella, G. (2004). *Monte carlo statistical methods*. Berlin: Springer Science & Business Media.

[CR65] Rosseel, Y. (2012). Lavaan: An R package for structural equation modeling. Journal of Statistical Software, **48**, 1-36. 10.18637/jss.v048.i02

[CR66] Rubin, D. B. (1976). Inference and missing data. *Biometrika,**63*(3), 581–592. 10.1093/biomet/63.3.581

[CR67] Schafer, J. L., & Graham, J. W. (2002). Missing data: Our view of the state of the art. *Psychological Methods,**7*(2), 147–177. 10.1037/1082-989X.7.2.147 12090408

[CR68] Serang, S., Zhang, Z., Helm, J., Steele, J. S., & Grimm, K. J. (2015). Evaluation of a Bayesian approach to estimating nonlinear mixed-effects mixture models. *Structural Equation Modeling: A Multidisciplinary Journal,**22*(2), 202–215. 10.1080/10705511.2014.937322

[CR69] Shi, D., DiStefano, C., Zheng, X., Liu, R., & Jiang, Z. (2021). Fitting latent growth models with small sample sizes and non-normal missing data. *International Journal of Behavioral Development,**45*(2), 179–192. 10.1177/0165025420979365 33664535 10.1177/0165025420979365 PMC7928428

[CR70] Smid, S. C., Depaoli, S., & Van De Schoot, R. (2020). Predicting a distal outcome variable from a latent growth model: ML versus Bayesian estimation. *Structural Equation Modeling: A Multidisciplinary Journal,**27*(2), 169–191. 10.1080/10705511.2019.1604140

[CR71] Song, X.-Y., & Lee, S.-Y. (2002). Analysis of structural equation model with ignorable missing continuous and polytomous data. *Psychometrika,**67*(2), 261–288. 10.1007/BF02294846

[CR72] Sterba, S. K. (2014). Fitting nonlinear latent growth curve models with individually varying time points. *Structural Equation Modeling: A Multidisciplinary Journal,**21*(4), 630–647. 10.1080/10705511.2014.919828

[CR73] Tanner, M. A., & Wong, W. H. (1987). The calculation of posterior distributions by data augmentation. *Journal of the American statistical Association,**82*(398), 528–540. 10.1080/01621459.1987.10478458

[CR74] Twisk, J., & de Vente, W. (2002). Attrition in longitudinal studies: How to deal with missing data. *Journal of Clinical Epidemiology,**55*(4), 329–337. 10.1016/S0895-4356(01)00476-0 11927199 10.1016/s0895-4356(01)00476-0

[CR75] van de Schoot, R., Depaoli, S., King, R., Kramer, B., Märtens, K., Tadesse, M. G., Vannucci, M., Gelman, A., Veen, D., Willemsen, J., et al. (2021). Bayesian statistics and modelling. *Nature Reviews Methods Primers,**1*(1), 1. 10.1038/s43586-020-00001-2

[CR76] van de Schoot, R., Sijbrandij, M., Depaoli, S., Winter, S. D., Olff, M., & Van Loey, N. E. (2018). Bayesian PTSD-trajectory analysis with informed priors based on a systematic literature search and expert elicitation. *Multivariate Behavioral Research,**53*(2), 267–291. 10.1080/00273171.2017.1412293 10.1080/00273171.2017.1412293 29324055

[CR77] van Buuren, S. (2018). *Flexible imputation of missing data*. Boca Raton: CRC Press.

[CR78] Veen, D., Egberts, M. R., Van Loey, N. E., & Van de Schoot, R. (2020). Expert elicitation for latent growth curve models: The case of posttraumatic stress symptoms development in children with burn injuries. *Frontiers in Psychology,**11*, 1197. 10.3389/fpsyg.2020.01197 10.3389/fpsyg.2020.01197 PMC7314932 32625139

[CR79] Veen, D., Stoel, D., Zondervan-Zwijnenburg, M., & van de Schoot, R. (2017). Proposal for a five-step method to elicit expert judgment. *Frontiers in Psychology,**8*, 2110. 10.3389/fpsyg.2017.02110 29259569 10.3389/fpsyg.2017.02110 PMC5723340

[CR80] Vehtari, A., Gelman, A., Simpson, D., Carpenter, B., & Bürkner, P.-C. (2021). Rank-normalization, folding, and localization: An improved R for assessing convergence of mcmc (with discussion). *Bayesian Analysis,**16*(2), 667–718. 10.1214/20-BA1221

[CR81] Wang, L., & McArdle, J. J. (2008). A simulation study comparison of Bayesian estimation with conventional methods for estimating unknown change points. *Structural Equation Modeling: A Multidisciplinary Journal,**15*(1), 52–74. 10.1080/10705510701758265

[CR82] Winter, S. D., & Depaoli, S. (2022). Sensitivity of Bayesian model fit indices to the prior specification of latent growth models. *Structural Equation Modeling: A Multidisciplinary Journal,**29*(5), 667–686. 10.1080/10705511.2022.2032078

[CR83] Wu, W., & Jia, F. (2021). Applying planned missingness designs to longitudinal panel studies in developmental science: An overview. *New Directions for Child and Adolescent Development,**2021*(175), 35–63. 10.1002/cad.20391 33470035 10.1002/cad.20391

[CR84] Wu, W., Jia, F., Rhemtulla, M., & Little, T. D. (2016). Search for efficient complete and planned missing data designs for analysis of change. *Behavior Research Methods,**48*(3), 1047–1061. 10.3758/s13428-015-0629-5 26170055 10.3758/s13428-015-0629-5

[CR85] Wu, W., West, S. G., & Hughes, J. N. (2008). Effect of retention in first grade on children’s achievement trajectories over 4 years: A piecewise growth analysis using propensity score matching. *Journal of Educational Psychology,**100*(4), 727–740. 10.1037/a0013098 19337582 10.1037/a0013098 PMC2662684

[CR86] Zondervan-Zwijnenburg, M., Peeters, M., Depaoli, S., & van de Schoot, R. (2017). Where do priors come from? applying guidelines to construct informative priors in small sample research. *Research in Human Development,**14*(4), 305–320. 10.1080/15427609.2017.1370966

